# Immunomodulatory and Metabolic Effects of Ginsenosides Rg1/Rb1 in IBD, RA, and MASLD: Targeting Gut Microbiota–Autoimmunity Axis

**DOI:** 10.3390/microorganisms14102246

**Published:** 2026-10-03

**Authors:** Xin Mao, Zongxin Shao, Min Gao, Weichen Wang, Yichen Jiang, Wenzhe Si, Yanyan Shi

**Affiliations:** 1 Research Centre of Clinical Epidemiology, Peking University Third Hospital, Beijing 100191, China; 2410108322@stu.pku.edu.cn (X.M.); 2210122508@stu.pku.edu.cn (Z.S.); 2110301346@bjmu.edu.cn (M.G.); 2510122516@stu.pku.edu.cn (W.W.); yorkcy2002@163.com (Y.J.); 2 Peking University Health Science Center, Peking University, Beijing 100191, China; 3 The First Affiliated Hospital, Zhejiang University School of Medicine, Hangzhou 311121, China; 4 Department of Laboratory Medicine, Key Laboratory of Cardiovascular Molecular Biology and Regulatory Peptides of National Health Commission, State Key Laboratory of Vascular Homeostasis and Remodeling, Peking University Third Hospital, Beijing 100191, China

**Keywords:** ginsenosides, metabolic diseases, immune system diseases, polypharmacology, gastrointestinal microbiome, network pharmacology

## Abstract

*Panax ginseng* Meyer has been commonly used as a traditional herbal medicine in East Asia for millennia. Ginsenosides Rg1 and Rb1 are the primary pharmacologically active ingredients and exhibit broad therapeutic efficacy against immunometabolic disorders, including inflammatory bowel disease (IBD), metabolic dysfunction-associated steatotic liver disease (MASLD), and rheumatoid arthritis (RA). To summarize the protective roles of these ginsenosides and investigate their underlying mechanisms, we integrated literature evidence with network pharmacology and gut microbiota–host gene association analyses. Published experimental studies have identified multiple mechanisms by which Rg1 and Rb1 ameliorate IBD, MASLD, and RA, including modulation of the phosphoinositide 3-kinase (PI3K)/Akt, AMP-activated protein kinase (AMPK), and peroxisome proliferator-activated receptor γ (PPARγ)/nuclear factor kappa-B (NF-κB) pathways, as well as direct toll-like receptor 4 (TLR4) binding and aryl hydrocarbon receptor (AHR)-mediated transcriptional regulation. Our network pharmacology analysis predicted target overlap among IBD, MASLD, and RA, prioritizing *TNF*, *NFKB1*, *PPARG*, and *MAPK1* as putative hubs. Our gut microbiota–host gene association analysis identified conserved microbial taxa sharing host gene targets with Rg1 and Rb1, such as *Akkermansia* and *Faecalibacterium*. Integrating these computational findings with published evidence, we propose that ginsenoside-associated shifts in these taxa may be linked to modulation of host signaling pathways, though causal relationships require experimental validation. This review summarizes the convergent mechanisms of Rg1 and Rb1 in ameliorating barrier-immunity-metabolism dysfunction across the gut-liver-joint axis. We also highlight the translational gap between preclinical research and clinical practice. This integrated analysis provides a novel research paradigm for understanding Rg1 and Rb1 as multi-target therapeutics for complex immunometabolic diseases.

## 1. Introduction

Immunometabolic diseases are chronic, complex disorders driven by the reciprocal regulation of immune dysregulation and metabolic imbalance. Inflammatory bowel disease (IBD), metabolic dysfunction-associated steatotic liver disease (MASLD), and rheumatoid arthritis (RA) represent three prevalent and clinically significant phenotypes characterized by persistent low-grade inflammation, metabolic dysfunction, and immune system disturbances [1,2,3]. These shared pathological features contribute to disease progression and therapeutic resistance. Metaflammation, defined as chronic metabolically driven inflammation, serves as a central pathogenic mechanism connecting the onset and clinical outcomes of these conditions [4]. The escalating global burden of immunometabolic diseases constitutes a significant public health concern. According to recent data, IBD affects approximately 5 million people worldwide, necessitating long-term immunomodulatory therapy to manage intestinal inflammation [5]. Similarly, MASLD, a nomenclature recently adopted to replace non-alcoholic fatty liver disease (NAFLD), represents the most common chronic liver disease. Epidemiological studies show a high concordance between the two definitions, with about 99% of individuals diagnosed with NAFLD also meeting the criteria for MASLD. It exhibits a strong association with obesity and type 2 diabetes and can progress to severe hepatic damage, including steatohepatitis, fibrosis, cirrhosis, and even liver cancer, thereby contributing significantly to global liver-related mortality and disease burden [6]. Furthermore, RA currently affects over 17 million individuals globally. With prevalence rates projected to rise, there is an urgent need for novel therapeutic strategies that address the underlying immunometabolic dysfunctions, as current standard-of-care treatments frequently fail to confer sustained remission or halt disease progression [7].

Despite distinct target organs and clinical manifestations, IBD, MASLD, and RA converge on fundamental etiological features, specifically chronic inflammation, immune dysfunction, and metabolic dysregulation. IBD is characterized by impaired intestinal barrier function, aberrant immune responses, and decreased microbial diversity, which collectively lead to chronic intestinal inflammation [8]. Accumulating evidence suggests that intestinal dysbiosis and barrier dysfunction may affect hepatic inflammation and metabolic homeostasis via the gut-liver axis [9]. MASLD, which manifests as hepatic steatosis associated with metabolic syndrome and insulin resistance, can advance from simple steatosis to stages involving hepatocyte injury, inflammation, and potentially liver fibrosis or cirrhosis. However, curative pharmacological options remain absent [10]. In RA, autoimmunity and inflammation within synovial tissues result in cartilage and bone destruction, while systemic inflammatory effects increase the risk of comorbidities such as cardiovascular disease [11,12]. Emerging evidence supports the concept of a gut-joint axis in RA, linking gut microbiota alterations to systemic immune dysregulation and joint inflammation [13]. Given their shared pathophysiology, it is critical to explore therapeutic strategies capable of dual modulation of the immune and metabolic components of these diseases.

Current pharmacological interventions, while capable of transiently alleviating inflammatory symptoms, are often ineffective in halting disease progression and are frequently associated with adverse effects [14]. These limitations have stimulated interest in alternative or complementary therapeutic approaches that modulate both immune and metabolic pathways.

Traditional Chinese medicine (TCM) has gained increasing attention as a promising approach for managing immunometabolic diseases [15]. Based on holistic philosophy and syndrome differentiation, TCM offers a unique perspective on disease prevention and treatment. Rather than targeting an isolated molecular pathway, TCM employs a multi-target strategy that coordinates signaling networks, metabolic processes, and cellular functions to achieve systemic intervention [16]. A pivotal mechanism underlying the therapeutic efficacy of TCM involves the modulation of gut microbiota. This modulation not only enhances intestinal barrier integrity but also reduces metabolic endotoxemia and inflammation, thereby facilitating the restoration of metabolic homeostasis [17].

Among TCM remedies, *Panax ginseng* Meyer is one of the most valued medicinal plants, widely used in East Asia for treating a variety of ailments. Modern pharmacological research has confirmed that *P. ginseng* exhibits diverse biological activities, including immunomodulation, regulation of glucose and lipid metabolism, and enhancement of host resilience. These effects are primarily attributed to ginsenosides, the principal bioactive saponins of ginseng, particularly the protopanaxatriol Rg1 and protopanaxadiol Rb1, which are the most abundant and bioactive constituents, known for their diverse bioactivities including anti-inflammatory effects, metabolic regulation [18]. Recent experimental and clinical studies indicate that ginsenosides exert beneficial effects on immunometabolic diseases, including modulation of immune cell activity, regulation of inflammatory cytokines, and modulation of energy metabolism pathways [19,20].

However, the precise molecular targets and core regulatory mechanisms of ginsenosides within the immunometabolic network remain insufficiently elucidated. This knowledge gap represents a major bottleneck limiting clinical translation and drug development [21]. This study provides a comprehensive review of the pharmacological effects and mechanisms of ginsenosides Rg1 and Rb1 in representative immunometabolic diseases, including IBD, MASLD, and RA. In addition, network pharmacology was employed to predict potential molecular targets, offering a theoretical framework for understanding their multi-target regulation of immunometabolic homeostasis. Furthermore, we conducted a gut microbiota–host gene association analysis to identify key microbial taxa and their associated host genes, providing complementary insights into microbiota-mediated mechanisms of ginsenoside action. Moreover, the current status of clinical trial registrations for Rg1 and Rb1 is reviewed to highlight ongoing efforts in translating these findings into clinical applications.

## 2. Methods

### 2.1. Study Selection Criteria

This review was based on a comprehensive literature search conducted in PubMed, Embase, and Web of Science. The search strategy combined MeSH terms and keywords related to “Inflammatory Bowel Disease”, “Colitis, Ulcerative”, “Crohn Disease”, “Non-alcoholic Fatty Liver Disease”, “Metabolic Dysfunction-Associated Steatohepatitis”, “Arthritis, Rheumatoid”, “ginsenoside Rg1”, and “ginsenoside Rb1”. No publication date restrictions were applied and the search was finalized in December 2025. The review considered experimental, and mechanistic studies addressing the pharmacological effects and mechanisms of ginsenosides Rg1 and Rb1, including their immunomodulatory, metabolic, and barrier-protective roles, as well as alterations in gut microbiota composition and host–microbe interactions. Because direct evidence simultaneously linking specific ginsenoside metabolites, gut microbiota, and therapeutic outcomes in MASLD and RA remains limited, particularly for microbiota-mediated mechanisms, relevant studies from immunology, endocrinology, microbiology, and host–microbiome research were also included when they provided biologically plausible insights into the proposed gut microbiota–autoimmunity axis. Accordingly, the available evidence was narratively integrated to organize biologically plausible relationships and identify testable hypotheses, rather than to establish a definitive causal pathway for each disease. In addition to the literature review, network pharmacology analyses were performed to predict potential targets of Rg1 and Rb1 using databases such as SwissTargetPrediction, PharmMapper, and Batman-TCM, and a gut microbiota–host gene association analysis was conducted via the gutMGene database to identify key microbial taxa and shared host genes. Because this study was not designed as a systematic review, no formal PRISMA-based study selection process, meta-analysis, or risk-of-bias assessment was performed.

### 2.2. The Methods of Network Pharmacology Analysis

This study employed network pharmacology to elucidate the molecular mechanisms underlying the therapeutic effects of ginsenosides.

Potential therapeutic targets for IBD, MASLD, and RA were retrieved from the GeneCards database (https://www.genecards.org/, accessed on 15 September 2025). The keywords “inflammatory bowel disease,” “nonalcoholic fatty liver disease,” and “rheumatoid arthritis” were used for retrieval, with the species restricted to *Homo sapiens*. All disease targets were converted to standard gene symbols via the UniProt database, and duplicate entries were removed after identifier normalization. For each disease, targets were ranked by Relevance score. IBD and RA each returned more than 2000 targets and were truncated at the top 2000; MASLD returned 1190 targets in total and was therefore included in full. The corresponding Relevance scores at the truncation point were 18.35 (IBD, 2000th), 19.39 (MASLD, 1190th) and 2.72 (RA, 2000th). Complete target lists are provided in Supplementary Table S1 (Table S1a,b).

Using ginsenoside Rg1 (PubChem CID: 441923) and Rb1 (PubChem CID: 9898279) as query molecules, potential target proteins were obtained from three drug target prediction databases: (1) SwissTargetPrediction (http://www.swisstargetprediction.ch/, accessed on15 September 2025); (2) PharmMapper (https://www.lilab-ecust.cn/pharmmapper/, accessed on 15 September 2025); and (3) BATMAN-TCM (http://bionet.ncpsb.org.cn/batman-tcm/, accessed on 15 September 2025). The species was restricted to *Homo sapiens* for all databases. The predicted results from the three databases were merged, and duplicate targets were removed.

Using an online bioinformatics platform (http://www.bioinformatics.com.cn; accessed on 15 September 2025), the targets of Rg1 and Rb1 were respectively intersected with disease targets to obtain overlapping targets. These overlaps were visualized as Venn diagrams.

The overlapping targets were imported into the STRING database (version 12.0; https://string-db.org/; accessed on 15 September 2025). *Homo sapiens* was chosen as the species and all active interaction sources were selected including textmining, experimentally determined, curated databases, co-expression, gene neighborhood, gene fusions, co-occurrence, and protein homology. A minimum required interaction score of 0.4 was used to construct the protein–protein interaction (PPI) network. In the network, nodes represent proteins, and edges indicate protein–protein interactions. Node colors denote functional clustering, which is automatically assigned by the built-in clustering algorithm in STRING. Proteins with the same color belong to the same functional module, suggesting their potential involvement in similar biological processes or signaling pathways. Edge colors represent the types of evidence supporting each interaction: green for gene neighborhood, red for gene fusions, blue for gene co-occurrence, yellow for textmining, purple for experimentally determined, cyan for curated databases, black for co-expression and light purple for protein homology; edges with multiple colors indicate that the interaction is supported by multiple lines of evidence. After excluding disconnected targets, the results were visualized and analyzed using the Cytoscape software (version 3.10.3).

The network analyzer plugin cytoHubba (version 0.1) in Cytoscape was used to calculate five topological parameters for each node in the network: MNC (Maximum Neighborhood Component), Degree, EPC (Edge Percolated Component), Betweenness, and Closeness. Nodes were ranked in descending order according to each of the five parameters, and the top 30 nodes from each ranking were selected. The intersection of the five sets of top-30 nodes was defined as the hub targets.

### 2.3. Gut Microbiota–Host Gene Association Analysis

Using the Gut Microbe-Gene database (gutMGene, http://www.gutmgene.org/, accessed on 16 October 2025), main microbial taxa sharing host gene targets with Rg1 and Rb1 were retrieved. Through the database’s search module, metabolites associated with these gut microbes were obtained. The potential targets of these microbial metabolites were predicted using the Similarity Ensemble Approach (SEA, https://sea.bkslab.org/, accessed on 21 October 2025). The top 10 targets with the highest prediction scores for each metabolite were selected (including all if less than 10). If the substance was not included in SEA, the target results from TargetNet (http://targetnet.scbdd.com/home/targets/, accessed on 27 October 2025) were used as a supplement. Finally, a “Drug-Host Target-Gut Microbiota-Microbial Metabolites-New Host Target” network was constructed using Cytoscape. The resulting network integrates relationships of three distinct evidence levels, which are explicitly distinguished throughout the manuscript. First, experimentally demonstrated interactions reported in the literature (e.g., intervention studies using FMT, gnotobiotic models or metabolite supplementation). Second, database-derived associations retrieved from gutMGene, in which a microbial taxon and a ginsenoside share a host gene target. Third, computationally predicted interactions generated by SEA or TargetNet. It should be emphasized that a shared host gene between a ginsenoside target and a microbial taxon indicates target overlap only; it does not demonstrate that Rg1 or Rb1 alters the abundance or activity of that taxon, nor that the microbial metabolites retrieved from the database mediate the therapeutic response.

Venn diagrams (panels a and d) were generated using an online platform for data analysis and visualization (https://www.bioinformatics.com.cn). Protein–protein interaction networks, hub-target subnetworks, and gut microbiota–host gene association networks (panels b, c, e, and f) were constructed and visualized in Cytoscape (version 3.10.3; Cytoscape Consortium, San Diego, CA, USA).

## 3. Pharmacological Effects and Mechanisms of Ginsenosides Rg1 and Rb1 in IBD

IBD, including ulcerative colitis and Crohn’s disease, is a chronic idiopathic inflammation of the gastrointestinal tract. The pathogenesis of IBD is multifactorial, involving oxidative stress, intestinal inflammation, and gut microbiota dysbiosis [22]. Studies have indicated that ginsenosides alleviate intestinal pathology through barrier-protective, antioxidant, anti-inflammatory, and immunomodulatory mechanisms (Table 1) [23].

### 3.1. Effects and Mechanisms of Ginsenoside Rg1 Against IBD

Ginsenoside Rg1 exhibits significant therapeutic efficacy in IBD through preserving intestinal barrier integrity, modulating immune responses, and exerting anti-inflammatory effects. As mucosal barrier disruption is a hallmark of IBD, Rg1 restores barrier integrity by upregulating tight junction proteins such as zonula occludens-1 (ZO-1) and repairing ultrastructure in DSS-induced colitis models. It also reduces pro-inflammatory cytokines like TNF-α and IFN-γ while increasing IL-4 [24], and protects epithelial cells by regulating the p38 MAPK/NLRP3 inflammasome pathway, reducing oxidative stress, and suppressing pro-apoptotic proteins such as Bax and Caspase-3 [25,31]. Furthermore, Rg1 strengthens intestinal barrier defense by stimulating intestinal epithelial cell proliferation and migration and increasing mucus layer secretion and thickness [32].

Immunologically, Rg1 regulates the differentiation of T-cells to restore immune tolerance and suppress excessive immune activation. It directly inhibits the PI3K/Akt signaling pathway and upregulates negative regulators such as PTEN, SOCS1, and SOCS3. This regulation leads to a reduction in pro-inflammatory T follicular helper (Tfh) subsets (including Tfh9 and Tfh17) and an expansion of T follicular regulatory (Tfr) and regulatory T (Treg) cells. Consequently, Rg1 corrects aberrant B-cell activation and antibody responses, thereby improving clinical and histopathological outcomes of dextran sodium sulfate (DSS)-induced colitis [26]. Similarly, it reduces the population of PD-1^+^ and PD-L1^+^ Treg cells to normalize the balance of memory Tfh cells. Additionally, Rg1 plays a critical role in macrophage polarization, promoting the conversion from a pro-inflammatory M1 phenotype to an anti-inflammatory M2 phenotype, which is a fundamental immunometabolic mechanism in IBD pathogenesis and resolution [33]. This phenotypic switch not only inhibits inflammatory pathways but also enhances tissue repair, consequently alleviating colonic inflammation and facilitating the restoration of intestinal homeostasis in IBD mice [27].

In summary, ginsenoside Rg1 exerts dual protective effects in IBD models, reinforcing intestinal barrier function and rebalancing the immune microenvironment. Its ability to modulate T-cell subsets and macrophage polarization, combined with barrier-protective effects, supports mucosal healing and resolves chronic inflammation.

### 3.2. Effects and Mechanisms of Ginsenoside Rb1 Against IBD

Ginsenoside Rb1 demonstrates comprehensive therapeutic potential by addressing several key pathological features of IBD, including epithelial barrier disruption, inflammation, oxidative stress, and microbiota dysbiosis. Rb1 plays a crucial role in restoring intestinal barrier integrity by enhancing the expression of tight junction proteins (Occludin and ZO-1) as well as the adherens junction protein E-cadherin, which strengthens the epithelial layer and reduces intestinal permeability. In DSS-induced colitis models, Rb1 treatment significantly improves the expression of these proteins and ameliorates histological damage, underscoring barrier restoration as a primary mechanism of action [28].

In addition, Rb1 promotes epithelial cell migration and wound healing through the activation of ERK and Rho signaling pathways [34]. It also mitigates inflammation-induced epithelial injury by modulating the Hrd1-associated endoplasmic reticulum stress pathway, thereby maintaining cellular protein homeostasis [29]. In terms of inflammatory signaling, Rb1 inhibits the NF-κB pathway by preventing IRAK-1 activation and regulating VDR and PPARγ transcriptional activity. This ensures coordinated control of inflammation and barrier maintenance in experimental colitis [28,30]. Furthermore, Rb1 exerts antioxidant effects by activating the PI3K/Akt/Nrf2 pathway, which enhances expression of antioxidant enzymes like HO-1 and NQO1 [35]. Although primarily demonstrated in LPS-induced systemic inflammation models lacking IBD-specific features, the ability of Rb1 to inhibit the TLR4/NF-κB axis suggests potential utility in mitigating the innate immune-driven cytokine storms (e.g., TNF-α, IL-6, and IL-1β) [36]. These findings suggest that targeting the TLR4 pathway with Rb1 could be a strategy to mitigate excessive inflammation in IBD [37].

Given its combined effects on barrier repair, immune modulation, and oxidative stress alleviation, Rb1 represents a multifaceted preclinical therapeutic approach for IBD, whereas its efficacy and safety in humans still demand further exploration through clinical trials.

### 3.3. Network Pharmacology Analysis of Rg1 and Rb1 in IBD

Using the GeneCards disease target database, 2000 potential therapeutic targets for IBD were identified. Concurrently, based on drug target databases including the Swiss Target Prediction platform, PharmMapper, and Batman-TCM, 333 and 320 putative target proteins for ginsenosides Rg1 and Rb1 were retrieved, respectively.

**Figure 1:**
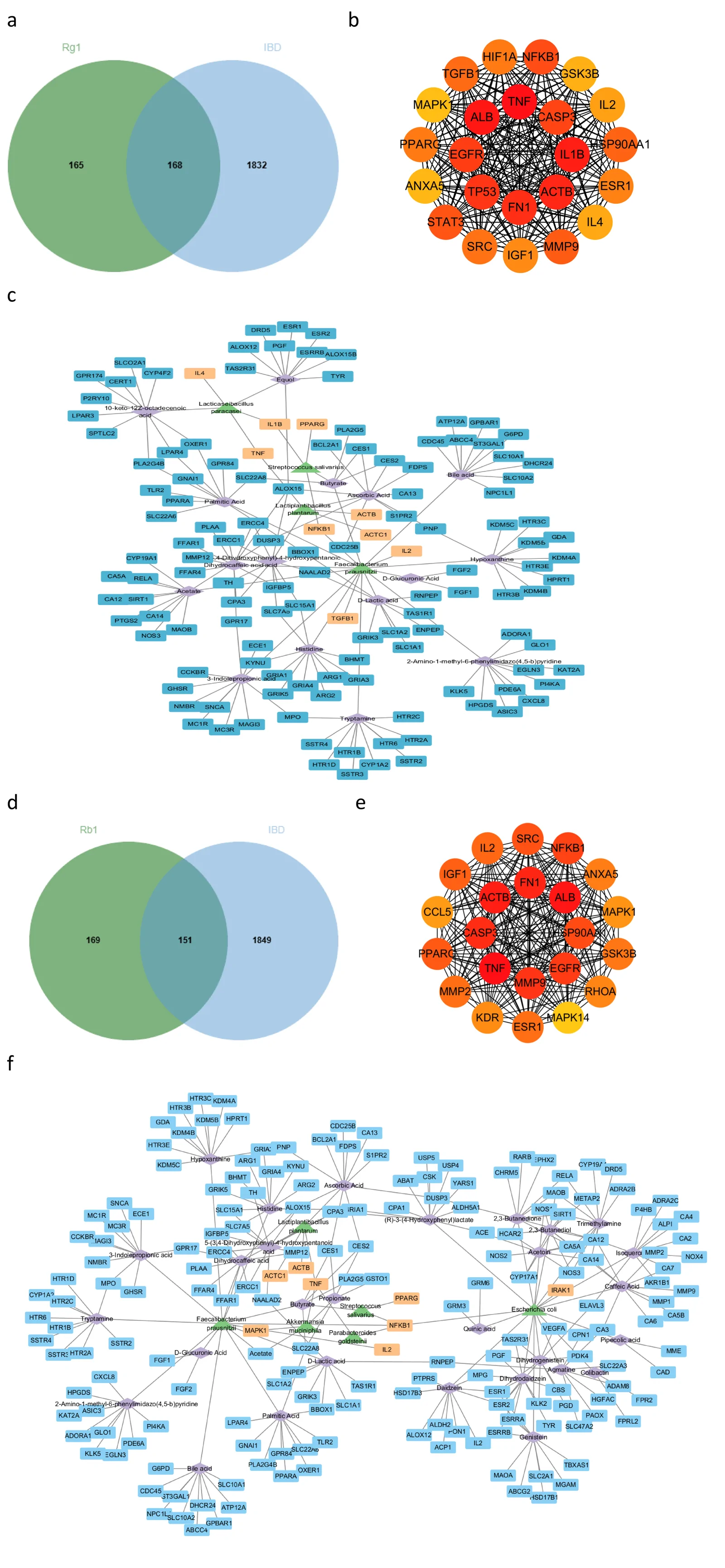
Prediction of therapeutic targets of ginsenoside Rg1 and Rb1 for IBD and gut microbiota–host gene association analysis for Rg1 and Rb1. These networks are hypothesis-generating and require experimental validation. (**a**) Venn diagram illustrating overlapping genes between Rg1 and IBD targets. (**b**) Identification of 23 predicted hub targets of Rg1 for IBD based on topological ranking (MNC, Degree, EPC, Betweenness, and Closeness). (**c**) Green triangles represent microbial taxa, orange rectangles symbolize host genes, purple diamonds indicate metabolites, blue rectangles denote microbial metabolite targets, and edges depict interaction relationships. (**d**) Venn diagram illustrating overlapping genes between Rb1 and IBD targets. (**e**) Identification of 22 predicted hub targets of Rb1 for IBD based on topological ranking (MNC, Degree, EPC, Betweenness, and Closeness). (**f**) Green triangles represent microbial taxa, orange rectangles symbolize host genes, purple diamonds indicate metabolites, blue rectangles denote microbial metabolite targets, and edges depict interaction relationships.

For Rg1, a Venn diagram revealed 168 overlapping targets with IBD (Figure 1a). These targets were submitted to the STRING database to analyze their interaction relationships. The output was mapped to a PPI network using Cytoscape software, comprising 168 nodes and 2827 edges in total (Figures S1 and S2). By ranking the overlapping targets according to MNC, Degree, EPC, Betweenness, and Closeness, and selecting the top 30 targets from each ranking to take the intersection, we identified 23 predicted hub targets associated with ginsenoside Rg1 in the treatment of IBD (Figure 1b). Likewise, 151 potential targets of Rb1 were identified (Figure 1d), yielding a PPI network with 150 nodes and 2060 connections (Figures S3 and S4). Subsequent screening yielded 22 predicted hub targets for Rb1 (Figure 1e).

To integrate host-microbiome interactions, we analyzed the gutMGene to identify microbial taxa sharing host gene targets with Rg1 and Rb1. Through the search module of the database, metabolites associated with these gut microbes were obtained. For Rg1, four bacterial taxa, including *Streptococcus salivarius*, *Faecalibacterium prausnitzii*, *Lacticaseibacillus paracasei*, and *Lactiplantibacillus plantarum*, were associated with the following shared host genes: *PPARG*, *IL1B*, *NFKB1*, *IL2*, *TGFB1*, *IL4*, *TNF*, *ACTC1*, and *ACTB* (Figure 1c). Further analysis of the database revealed that these strains were reported to produce 16 unique metabolites including butyrate, bile acid, and equol. For Rb1, six key bacterial taxa, including *Lactiplantibacillus plantarum*, *Streptococcus salivarius*, *Faecalibacterium prausnitzii*, *Akkermansia muciniphila*, *Escherichia coli*, and *Parabacteroides goldsteinii*, were linked to host genes such as *TNF*, *ACTC1*, *ACTB*, *PPARG*, *NFKB1*, *IL2*, *MAPK1*, and *IRAK1* (Figure 1f). These strains were reported to produce 30 unique metabolites including butyrate, bile acid, and 2,3-butanedione. SEA (and TargetNet when necessary) was subsequently employed to predict potential targets for these microbial metabolites, on this basis we constructed a multidimensional network linking predicted and database-derived relationships among ginsenosides, gut microbiota, metabolites and host genes.

## 4. Pharmacological Effects and Mechanisms of Ginsenosides Rg1 and Rb1 in MASLD

MASLD is one of the most prevalent chronic liver diseases worldwide, affecting approximately 38% of the global population and imposing a substantial burden on public health systems [38,39]. The prevailing “multiple-hit” hypothesis in MASLD research posits that the disease develops through the integrated contributions of diverse factors, such as insulin resistance, immune dysregulation, adipokine activity, dietary components, gut microbiota alterations, and genetic and epigenetic influences [40]. These pathogenic processes drive disease progression from simple steatosis to metabolic dysfunction-associated steatohepatitis (MASH), fibrosis, cirrhosis, and ultimately hepatocellular carcinoma [41,42]. Current management strategies primarily rely on lifestyle modification, particularly sustained weight reduction through dietary intervention and physical activity, to ameliorate hepatic steatosis and improve metabolic parameters. Emerging pharmacological agents targeting metabolic, inflammatory, and fibrotic pathways, such as FXR agonists, PPAR modulators, and GLP-1 receptor agonists, are currently undergoing clinical investigation [43]. Despite these advances, effective and widely applicable therapies for MASLD remain limited, underscoring the necessity for novel therapeutic approaches. In this context, ginsenosides have attracted increasing attention as potential hepatoprotective agents. Accumulating evidence suggests that the beneficial effects of ginsenosides are mediated by the coordinated regulation of hepatic lipid metabolism, attenuation of oxidative stress and inflammatory signaling pathways, and modulation of gut microbiota composition and function (Table 2) [44,45]. Notably, ginsenosides Rg1 and Rb1 regulate the gut-liver axis by restoring bile acid (BA) homeostasis via microbiota remodeling. By enriching genera such as *Lachnoclostridium* and *Blautia*, these compounds modulate TGR5 and FXR/CYP7A1 signaling to enhance thermogenesis and attenuate hepatic lipid accumulation, underscoring their relevance to MASLD metabolism [46,47].

### 4.1. Effects and Mechanisms of Ginsenoside Rg1 Against MASLD

Excessive hepatic lipid accumulation is a hallmark of MASLD. Increasing evidence indicates that ginsenoside Rg1 modulates hepatic lipid metabolism and reduces lipid deposition through multiple mechanisms, thereby exerting hepatoprotective effects and improving metabolic homeostasis. In hepatocyte models, Rg1 significantly reduces intracellular triglyceride accumulation in free fatty acid-treated HepG2 cells by suppressing key regulators of lipogenesis and fatty acid uptake, including SREBP-1c, FASN, CD36, FATP2/5, and PPARγ. Concomitantly, Rg1 upregulates genes involved in fatty acid oxidation and lipid export, such as PPARα, CPT1, ACOX1, MTTP, and ApoB100 [54]. This dual regulatory pattern shifts hepatic lipid handling away from synthesis and storage toward oxidation and secretion, thereby limiting triglyceride deposition. Consistently, in MASLD rat models, Rg1 markedly decreases hepatic lipid deposition and serum triglyceride levels. Mechanistically, Rg1 suppresses SREBP-1c expression and its downstream lipogenic program while enhancing the expression of β-oxidation-related genes, including PPARα, CPT1, CPT2, and CYP7A, indicating enhanced fatty acid catabolism [44]. Activation of AMP-activated protein kinase (AMPK) appears to represent a central upstream event mediating these metabolic effects. In both hepatocyte and adipose tissue models, Rg1-induced AMPK activation inhibits lipogenesis and stimulates fatty acid oxidation, consistent with the pivotal role of AMPK in maintaining anabolic-catabolic balance [54,55].

Beyond its direct hepatic actions, Rg1 regulates systemic lipid metabolism in a manner that indirectly alleviates hepatic lipid burden. Rg1 inhibits early adipocyte differentiation through activation of CHOP10, thereby limiting adipose tissue expansion and lipid storage at an early stage. In mature adipocytes and high-fat diet (HFD)-fed mice, Rg1 further suppresses adipogenic programs while enhancing lipolysis via AMPK activation, leading to reduced peripheral lipid accumulation [55]. Moreover, Rg1 facilitates the browning of white adipose tissue, characterized by increased UCP1 expression and enhanced mitochondrial activity, leading to elevated energy expenditure and restricting lipid spillover to the liver [56]. These effects reduce lipid flux from adipose tissue to the liver and contribute to the maintenance of hepatic lipid homeostasis.

Rg1 also exerts protective effects against MASLD by attenuating oxidative stress and inflammatory signaling. In hepatocyte models, Rg1 alleviates palmitic acid-induced steatosis and inflammatory responses through modulation of the AMPK/NF-κB axis [45]. In vivo studies further demonstrate that Rg1 suppresses hepatic inflammation by downregulating pro-inflammatory mediators [48]. Mechanistic evidence implicates FOXO1 as a critical regulatory node linking lipid metabolism and oxidative balance in Rg1-treated fatty liver models [50]. Rg1 also reduces lipid peroxidation, endoplasmic reticulum stress, and inflammasome activation, consequently preventing hepatocellular injury and inflammatory amplification [57]. These antioxidant and anti-inflammatory effects converge to alleviate insulin resistance and interrupt lipotoxic stress, ultimately limiting downstream hepatocyte apoptosis. Rg1 attenuates diet-induced hepatic insulin resistance by suppressing NF-κB–dependent expression of inflammatory cytokines, including IL-1β, IL-6, and TNF-α, thereby restoring IRS/PI3K/Akt signaling [48]. In addition, Rg1 exhibits potent anti-apoptotic effects by downregulating SGPL1, a key regulator of sphingolipid metabolism implicated in lipotoxic hepatocyte apoptosis [51]. Multi-omics and transcriptomic analyses further substantiate the broad regulatory capacity of Rg1, revealing extensive modulation of genes and metabolites associated with oxidative stress and inflammatory pathways. This indicates a global transcriptional reprogramming that enhances hepatocellular resilience [49,58].

As a major bioactive saponin of ginseng, Rg1 has demonstrated preclinical efficacy in MASLD models. However, it remains elusive whether these benefits are primarily mediated by the parent compound, its in vivo metabolites, or the coordinated actions of both. Collectively, elucidating the precise molecular forms and targets responsible for Rg1 activity will be essential for defining its mechanism of action and facilitating the development of Rg1-based therapeutic strategies for MASLD.

### 4.2. Effects and Mechanisms of Ginsenoside Rb1 Against MASLD

Ginsenoside Rb1, another major bioactive component of ginseng, exhibits pronounced hepatoprotective effects in MASLD through coordinated regulation of lipid metabolism, inflammatory signaling, and hepatocyte survival. At the level of systemic lipid flux, Rb1 reduces hepatic lipid overload by modulating adipose-liver crosstalk. In db/db obese mice, Rb1 upregulates perilipin expression in adipose tissue to stabilize lipid droplets and suppress excessive adipocyte lipolysis, consequently limiting the release of free fatty acids to the liver. This reduction in lipid influx is accompanied by improved hepatic insulin sensitivity and attenuation of steatosis [59]. Consistently, Rb1 enhances adiponectin secretion from adipocytes. Notably, conditioned media derived from Rb1-treated adipocytes markedly reduce triglyceride accumulation in hepatocytes, an effect that is largely abolished in the absence of adiponectin. This underscores the critical role of adiponectin as a mediator of the metabolic benefits induced by Rb1 [60].

Rb1 regulates hepatic lipid metabolism by activating AMPK signaling, thereby promoting fatty acid oxidation and suppressing de novo lipogenesis. In HFD-induced obese rats, Rb1 stimulation of hepatic AMPK is associated with increased oxidative capacity and a significant reduction in hepatic triglyceride content [61]. These findings highlight the integrative role of Rb1 in restoring metabolic flexibility in MASLD.

Increasing evidence indicates that Rb1 mitigates hepatic inflammation and oxidative stress by regulating key signaling pathways. In models of D-galactosamine/lipopolysaccharide (D-GalN/LPS)-induced acute liver injury, Rb1 markedly suppresses the TLR4/NF-κB signaling pathway, resulting in reduced phosphorylation of IκBα and decreased hepatic expression of pro-inflammatory cytokines, including TNF-α, IL-1β, and IL-6. Furthermore, Rb1 inhibits activation of the NLRP3 inflammasome primarily in Kupffer cells (KCs), which leads to reduced caspase-1 activity and diminished release of IL-1β and IL-18, thereby alleviating hepatocellular necrosis [62]. Given that pharmacological or genetic inhibition of NLRP3 functions to attenuate hepatic inflammation and fibrosis in experimental MASH models, targeting this pathway represents a central mechanism underlying the anti-inflammatory effects of Rb1 [63].

Oxidative stress denotes another critical pathological axis in MASLD, and Rb1 effectively restores hepatic redox homeostasis through AMPK-dependent regulation of mitochondrial function. Activation of AMPK enhances mitochondrial oxidative metabolism, reduces reactive oxygen species production, and prevents lipid peroxidation, thereby stabilizing hepatocellular membranes and improving antioxidant defense capacity [61]. Moreover, the upregulation of adiponectin by Rb1 further contributes to the suppression of oxidative stress and inflammatory signaling in hepatocytes, reinforcing its protective effects against diet-induced hepatic injury [60].

Regarding hepatocyte fate determination, Rb1 exerts dual regulatory effects on apoptosis and autophagic lipid degradation. Rb1 alleviates HFD-induced hepatocytic apoptosis through activation of the PPARγ signaling pathway. Upregulation of PPARγ reduces endoplasmic reticulum stress and inhibits apoptotic cascades, as evidenced by decreased expression of pro-apoptotic Bax and cleaved caspase-3, alongside increased Bcl-2 expression [52]. Additionally, Rb1 promotes autophagic clearance of lipid droplets via the miR-128/TFEB axis. Downregulation of miR-128 facilitates nuclear translocation of transcription factor EB (TFEB), enhancing lysosomal biogenesis and autophagic flux. This mechanism accelerates intracellular lipid degradation and alleviates hepatic steatosis [53].

Collectively, these discoveries demonstrate the comprehensive hepatoprotective effects of ginsenoside Rb1 in MASLD, involving integration of metabolic regulation, suppression of inflammation and oxidative stress, and preservation of hepatocyte integrity. This multidimensional mode of action positions Rb1 as a prospective therapeutic candidate for preventing disease progression.

### 4.3. Network Pharmacology Analysis of Rg1 and Rb1 in MASLD

To elucidate the molecular mechanisms underlying the therapeutic effects of ginsenosides, we performed a network pharmacology analysis. Using the GeneCards database, 1190 potential therapeutic targets for MASLD were predicted. Concurrently, 333 and 320 putative targets for ginsenosides Rg1 and Rb1, respectively, were retrieved from the SwissTargetPrediction, PharmMapper, and Batman-TCM databases.

**Figure 2:**
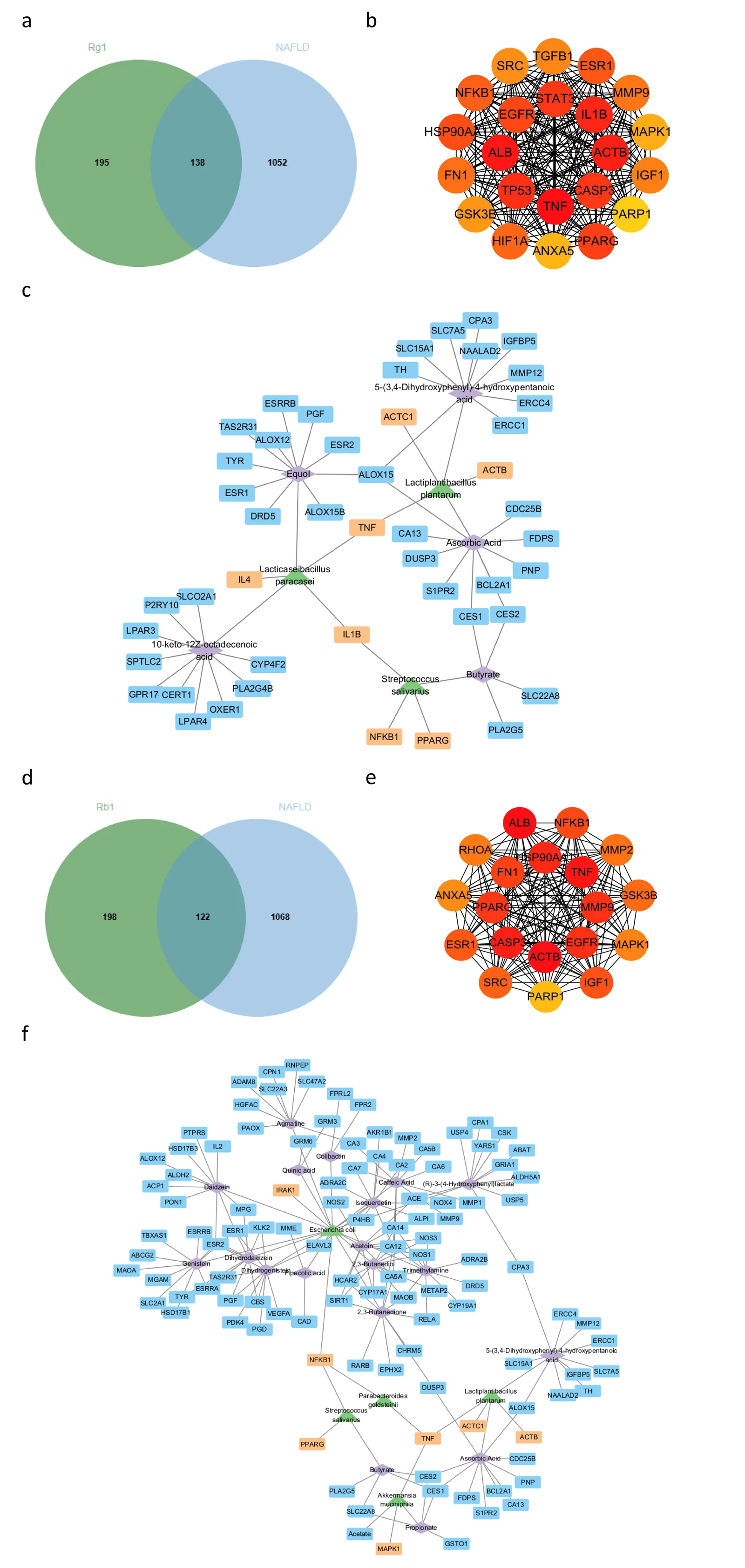
Prediction of therapeutic targets of ginsenoside Rg1 and Rb1 for MASLD and gut microbiota–host gene association analysis for Rg1 and Rb1. These networks are hypothesis-generating and require experimental validation. (**a**) Venn diagram showing the overlapping genes between ginsenoside Rg1 and MASLD targets. (**b**) Screening of 22 predicted hub targets of ginsenoside Rg1 for the treatment of MASLD based on ranking by MNC, Degree, EPC, Betweenness, and Closeness. (**c**) Green triangles represent microbial taxa, orange rectangles symbolize host genes, purple diamonds indicate metabolites, blue rectangles denote microbial metabolite targets, and edges depict interaction relationships. (**d**) Venn diagram showing the overlapping genes between ginsenoside Rb1 and MASLD targets. (**e**) Screening of 19 predicted hub targets of ginsenoside Rb1 for the treatment of MASLD based on ranking by MNC, Degree, EPC, Betweenness, and Closeness. (**f**) Green triangles represent microbial taxa, orange rectangles symbolize host genes, purple diamonds indicate metabolites, blue rectangles denote microbial metabolite targets, and edges depict interaction relationships.

For Rg1, a Venn diagram identified 138 overlapping targets between MASLD and Rg1 (Figure 2a). Analyzing interaction relationships in the STRING database, these targets were mapped to a PPI network using Cytoscape, comprising 138 nodes and 2364 edges (Figures S5 and S6). Topological analysis based on MNC, Degree, EPC, Betweenness, and Closeness identified 22 predicted hub targets associated with Rg1 treatment (Figure 2b). Applying the same methodology for Rb1, 122 potential targets were identified (Figure 2d), yielding a PPI network with 122 nodes and 1677 connections (Figures S7 and S8). Ultimately, 19 predicted hub targets for Rb1 were identified (Figure 2e).

Given the role of the gut microbiome in xenobiotic metabolism, we accessed the Gut Microbe-Gene database to identify microbial taxa sharing host gene targets with Rg1 and Rb1. For Rg1, we identified 3 key bacterial taxa: *Streptococcus salivarius*, *Lacticaseibacillus paracasei*, and *Lactiplantibacillus plantarum* (Figure 2c). They were associated with shared host genes including *PPARG*, *IL1B*, *NFKB1*, *IL4*, *TNF*, *ACTC1*, and *ACTB*. Further analysis revealed that these strains produced 5 unique metabolites including butyrate and equol. For Rb1, five bacterial taxa, including *Lactiplantibacillus plantarum*, *Streptococcus salivarius*, *Akkermansia muciniphila*, *Escherichia coli*, and *Parabacteroides goldsteinii*, were linked to host genes such as *TNF*, *ACTC1*, *ACTB*, *PPARG*, *NFKB1*, *MAPK1*, and *IRAK1* (Figure 2f). Further analysis revealed that these strains were reported to produce 20 unique metabolites including butyrate and 2,3-butanedione. The SEA was subsequently employed to predict potential targets for these microbial metabolites, constructing a multidimensional interaction network connecting ginsenosides, gut microbiota, metabolites, and host genes.

## 5. Pharmacological Effects and Mechanisms of Ginsenosides Rg1 and Rb1 in RA

RA is a chronic autoimmune disorder characterized by persistent synovial inflammation, progressive joint damage, and systemic bone loss, imposing a significant global burden on patients and healthcare systems [64]. The pathogenesis of RA is multifactorial, including genetic susceptibility, environmental and mucosal triggers, and profound dysregulation of innate and adaptive immune responses [65]. Skeletal involvement in RA manifests through three interrelated pathological processes: focal bone erosions at inflamed joints, periarticular osteopenia, and systemic osteoporosis. These processes are predominantly driven by disproportionate osteoclast differentiation and activity, orchestrated through inflammatory signaling cascades, particularly NFATc1-dependent pathways [66,67]. At the molecular and cellular levels, chronic synovitis, proinflammatory cytokine-mediated osteoclastogenesis, and metabolic reprogramming associated with immune dysfunction collectively contribute to bone destruction in RA [68].

Current therapeutic strategies for RA, comprising conventional and targeted disease-modifying antirheumatic drugs (DMARDs), biologic agents, and small-molecule inhibitors such as Janus kinase (JAK) inhibitors, effectively attenuate synovial inflammation while limiting structural joint damage and osteoporosis [64]. Recently, the gut-joint axis has been recognized as a pivotal determinant of RA pathogenesis, with gut microbiota dysbiosis playing an essential role in regulating immune homeostasis and skeletal metabolism [69]. Emerging therapeutic paradigms increasingly focus on targeting pathogenic immune pathways and modulating the gut microbiome, offering avenues for precision medicine-based management [13,70]. Studies indicate that ginsenosides Rg1 and Rb1 modulate key processes such as synovial inflammation, osteoclast differentiation, osteoblast maturation, and T-cell responses. The mechanisms through which Rg1 and Rb1 intersect with RA pathophysiology are examined below, integrating evidence from established RA models and from related bone, cartilage, and inflammatory models that capture discrete facets of RA pathophysiology (Table 3).

### 5.1. Effects and Mechanisms of Ginsenoside Rg1 Against RA

In RA, persistent synovial inflammation stimulates excessive osteoclast activation, impaired osteoblast differentiation, and disrupted bone remodeling. These factors cumulatively induce progressive focal bone erosion and systemic bone loss [66,82]. Accordingly, targeting both inflammatory signaling and skeletal remodeling is essential for preventing structural joint damage.

Ginsenoside Rg1 modulates bone resorption and osteogenesis, two key pathological processes in RA, through coordinated regulation of inflammatory responses, immune balance, and lymphatic function. At the level of bone resorption, Rg1 potently inhibits osteoclast differentiation and maturation. In RANKL-stimulated RAW264.7 macrophages, Rg1 significantly reduces the formation of multinucleated TRAP-positive osteoclasts and suppresses bone-resorption pit formation. This inhibition is accompanied by the downregulation of osteoclast-specific genes (e.g., Trap, Cathepsin K, Mmp9, Ctr) and key transcriptional regulators of osteoclastogenesis (e.g., c-Fos, c-Jun, NFATc1). These in vitro anti-resorptive effects are consistently observed in vivo. In collagen-induced arthritis (CIA) mice, Rg1 alleviates arthritis severity, attenuates synovial hyperplasia, and decreases the number of TRAP-positive osteoclasts in subchondral bone [71]. Furthermore, in TNF-transgenic mice, Rg1 improves lymphatic function by increasing smooth muscle cell coverage of collecting lymphatic vessels. The aforementioned process enhances lymphatic contractility and drainage, thereby markedly mitigating synovial inflammation, joint swelling, inflammatory cell infiltration, and bone erosion, alongside downregulated systemic levels of IL-6 and TNF-α [73].

In terms of osteogenesis and bone homeostasis, Rg1 maintains the dynamic balance of remodeling by directly enhancing osteogenic differentiation and regulating oxidative stress and inflammatory signaling pathways. Cell-based studies confirm that Rg1 activates the PI3K/Akt signaling pathway in a G protein-coupled estrogen receptor (GPER)-dependent manner to promote osteoblast maturation [75]. Meanwhile, Rg1 exerts prominent regulatory effects on joint inflammation and immune responses. In adjuvant-induced arthritis (AIA) rats, Rg1 activates PPARγ to sup-press NF-κB signaling, reduce pro-inflammatory cytokines, and limit inflammation-mediated bone loss [74]. In CIA mice, Rg1 directly modulates joint-resident immune responses by rebalancing T-cell subsets (reducing activated CD4^+^ T cells), decreasing local TNF-α and IL-6 levels, and preserving cartilage integrity, which ultimately reduces arthritis index scores [72].

Taken together, these findings illustrate that ginsenoside Rg1 mitigates the key pathological features of RA through integrated regulation of immune responses, inflammatory signaling, and bone remodeling. These polyvalent effects implicate Rg1 as a potent therapeutic agent for RA treatment.

### 5.2. Effects and Mechanisms of Ginsenoside Rb1 Against RA

Ginsenoside Rb1 was reported as early as 1997 to possess anti-arthritic properties mediated by integrated anti-inflammatory and immunoregulatory mechanisms. Specifically, Rb1 suppresses NF-κB inflammatory signaling to reduce production of pro-inflammatory cytokines such as TNF-α and IL-1β, thereby alleviating synovial inflammation and joint destruction in experimental RA models. In parallel, Rb1 enhances Treg responses, contributing to the restoration of immune tolerance and correction of autoimmune dysregulation [76]. Beyond anti-inflammation and immunomodulation, Rb1 targets key processes of cartilage degradation. It exhibits significant chondroprotective potential by inhibiting matrix metalloproteinase (MMP) expression and synovial fibroblast activation, key drivers of cartilage degradation in RA [77,83,84]. Additionally, Rb1 augments autophagy in synovial cells, a process that reduces inflammatory cytokine production and promotes tissue repair, further reinforcing its protective effects against joint injury [85].

In vitro, Rb1 effectively suppresses receptor activator of NF-κB ligand (RANKL)-induced osteoclastogenesis, underscoring a capacity to inhibit pathological bone resorption, a central feature of RA-mediated bone destruction. Mechanistically, Rb1 interferes with early osteoclast differentiation by modulating essential signaling cascades, including NF-κB activation and MAPK pathways, particularly JNK and p38, which are indispensable for the commitment and maturation of osteoclast precursors [81]. Further studies have identified the aryl hydrocarbon receptor (AHR)/PRELP/NF-κB axis as critical to the anti-resorptive effects of Rb1. As an AHR agonist, Rb1 upregulates the expression of proline/arginine-rich end leucine-rich repeat protein (PRELP), which serves as a decoy receptor for RANKL or interacts with RANK on osteoclast precursors. This interaction disrupts RANKL/RANK signaling and attenuates downstream NF-κB activation, ultimately suppressing osteoclast differentiation. Notably, the functional importance of AHR/PRELP/NF-κB axis has been validated in vivo, where it mediates the protective effects of Rb1 against bone loss in ovariectomized mouse models [78]. Although ovariectomy-induced bone loss arises from estrogen deficiency rather than autoimmune inflammation, both conditions converge on excessive osteoclast-mediated bone resorption, a process that drives focal bone erosions in RA.

Rb1 also exerts direct chondroprotective actions that counteract cartilage degeneration. In rat articular chondrocytes exposed to hydrogen peroxide-induced oxidative stress, Rb1 enhances cell viability and suppresses apoptosis through preservation of mitochondrial membrane potential, inhibition of cytochrome c release, and attenuation of caspase-3 activation [86]. Furthermore, Rb1 reduces extracellular matrix degradation by downregulating MMP-13, a key enzyme responsible for type II collagen breakdown. Mechanistic insights from osteoarthritis models indicate that these protective effects are at least mediated through suppression of Notch signaling, as evidenced by decreased levels of the Notch1 intracellular domain (NICD) [79]. This inhibition of collagen degradation bears particular relevance to RA pathogenesis. The proteolytic degradation of type II collagen generates remnant epitopes that serve as autoantigens to initiate or perpetuate the autoimmune response in RA [87]. While MMP-8 is implicated as the primary collagenase responsible for type II collagen cleavage in human RA, MMP-13 remains the dominant enzyme driving collagen degradation in murine models and human osteoarthritis [83]. Consequently, Rb1-mediated downregulation of MMP-13 provides a rationale for its potential to preserve the extracellular matrix and limit immunogenic collagen fragments generation in the inflammatory context of RA.

Besides, Rb1 exhibits potent anti-inflammatory and immunomodulatory activities relevant to RA pathogenesis. While direct evidence in RA-specific models is limited, findings from related inflammatory and autoimmune models suggest that Rb1 modulates pathways relevant to RA pathogenesis. In the LPS-induced inflammatory disease model, Rb1 modulates TLR4 dimerization, leading to suppression of NF-κB and MAPK signaling pathways. Consequently, Rb1 reduces the production of major pro-inflammatory cytokines, including TNF-α, IL-6, and IL-1β, which are central mediators in RA pathology [37]. In addition, Rb1 exerts immunomodulatory effects on adaptive immunity. In experimental autoimmune settings, Rb1 suppresses pathogenic B-cell activation and autoantibody production and shifts T helper cell responses away from pro-inflammatory Th1 and Th17 phenotypes. Given the well-established roles of autoreactive B cells and Th1/Th17 polarization in the initiation and perpetuation of RA, these findings suggest that Rb1 may exert disease-modifying effects through pathways highly relevant to autoimmune arthritis [80]. Nevertheless, further studies using RA-specific experimental models are necessary to confirm these potential effects.

In conclusion, while direct clinical evidence and RA-specific preclinical data remain limited, existing studies in related bone, cartilage, and inflammatory models provide mechanistic insights that inform the potential relevance of Rb1 to RA pathophysiology. Further research using RA-specific experimental models and rigorously designed clinical trials is warranted to validate its therapeutic potential.

### 5.3. Network Pharmacology Analysis of Rg1 and Rb1 in RA

This study employed network pharmacology to explore the molecular mechanisms underlying the therapeutic effects of ginsenosides in RA. We retrieved 2000 putative therapeutic targets for RA from the GeneCards database. Concurrently, we identified 333 and 320 potential protein targets for ginsenosides Rg1 and Rb1, respectively, utilizing the SwissTargetPrediction, PharmMapper, and Batman-TCM databases.

**Figure 3:**
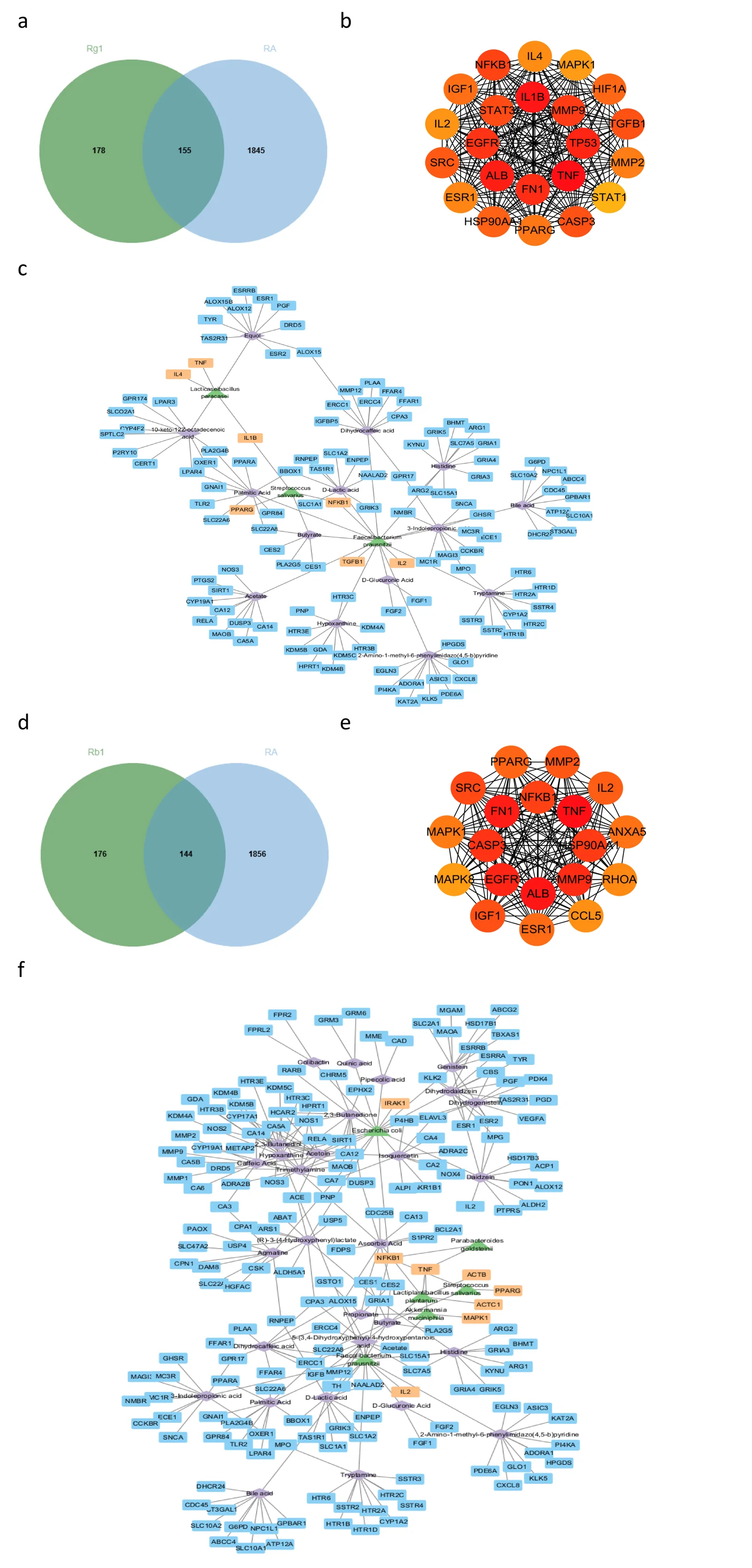
Prediction of therapeutic targets of ginsenoside Rg1 and Rb1 for RA and gut microbiota–host gene association analysis for Rg1 and Rb1. These networks are hypothesis-generating and require experimental validation. (**a**) Venn diagram showing the overlapping genes between ginsenoside Rg1 and RA targets. (**b**) Screening of 22 predicted hub targets of ginsenoside Rg1 for the treatment of RA based on ranking by MNC, Degree, EPC, Betweenness, and Closeness. (**c**) Green triangles represent microbial taxa, orange rectangles symbolize host genes, purple diamonds indicate metabolites, blue rectangles denote microbial metabolite targets, and edges depict interaction relationships. (**d**) Venn diagram showing the overlapping genes between ginsenoside Rb1 and RA targets. (**e**) Screening of 19 predicted hub targets of ginsenoside Rb1 for the treatment of RA based on ranking by MNC, Degree, EPC, Betweenness, and Closeness. (**f**) Green triangles represent microbial taxa, orange rectangles symbolize host genes, purple diamonds indicate metabolites, blue rectangles denote microbial metabolite targets, and edges depict interaction relationships.

For Rg1, construction of a Venn diagram revealed 155 overlapping targets with RA (Figure 3a). These targets were submitted to the STRING database to analyze their interaction relationships, and then the output was mapped to a PPI network using Cytoscape, encompassing 154 nodes and 2469 edges (Figures S9 and S10). Analysis according to MNC, Degree, EPC, Betweenness, and Closeness identified 22 predicted hub targets associated with ginsenoside Rg1 in the treatment of RA (Figure 3b). Similarly, for Rb1, 144 potential targets were identified (Figure 3d), and these were used to construct a PPI network containing 143 nodes and 1895 connections (Figures S11 and S12). Subsequent screening identified 19 predicted hub targets for Rb1 (Figure 3e).

To integrate host-microbiome interactions, we analyzed the gutMGene database to identify microbial taxa sharing host gene targets with Rg1 and Rb1. For Rg1, three key bacterial strains, including *Streptococcus salivarius*, *Faecalibacterium prausnitzii,* and *Lacticaseibacillus paracasei*, were linked to shared host genes such as *PPARG*, *IL1B*, *NFKB1*, *IL2*, *TGFB1*, *IL4*, and *TNF* (Figure 3c). Further analysis revealed that these strains were reported to produce 14 unique metabolites including butyrate, 3-indolepropionic acid, and bile acids. For Rb1, we identified 6 bacterial taxa sharing host genes with Rb1, including *Lactiplantibacillus plantarum*, *Streptococcus salivarius*, *Faecalibacterium prausnitzii*, *Akkermansia muciniphila*, *Escherichia coli*, and *Parabacteroides goldsteinii* (Figure 3f). These bacteria were associated with the following shared host genes: *TNF*, *ACTC1*, *ACTB*, *PPARG*, *NFKB1*, *IL2*, *MAPK1*, and *IRAK1*. Further analysis identified that these strains were reported to produce 30 unique metabolites including butyrate, 3-indolepropionic acid, and bile acids. Subsequently, SEA was employed to identify the top 10 potential targets for these metabolites.

## 6. Modulation of Gut Microbiota by Ginsenosides Rg1 and Rb1 in IBD, MASLD, and RA

Mounting evidence suggests that the therapeutic efficacy of ginsenosides Rg1 and Rb1 in immunometabolic diseases is not solely determined by their direct bioavailability, but by a dynamic, bidirectional interaction with the gut microbiota [88]. The gut microbiota functions as a biological transformer, metabolizing parent ginsenosides (particularly Rb1) into bioactive secondary saponins that are crucial for systemic absorption. In turn, ginsenosides act as modulators reshaping the microbial community to normalize the immune responses and metabolic homeostasis in chronic immunometabolic diseases [89,90]. These compounds favorably influence microbial composition by enriching beneficial taxa, such as *Lactobacillus* and *Akkermansia*, while reducing the abundance of potentially pathogenic or pro-inflammatory microorganisms. In IBD, Rg1 has been reported to restore microbial diversity and improve intestinal barrier function [27]. Conversely, direct evidence supporting microbiota-related effects of Rb1 remains limited. For MASLD and RA, the specific impacts of Rg1 and Rb1 on gut microbiota composition and function require further elucidation. Nevertheless, these findings suggest that these ginsenosides may contribute to immune and metabolic homeostasis through microbiota-associated mechanisms, warranting further investigation into their therapeutic potential for managing immunometabolic diseases via microbiota regulation [91].

### 6.1. Microbial Biotransformation and Bioavailability of Rg1 and Rb1

Considering the structural complexity and limited oral bioavailability of ginsenosides, delineating their pharmacokinetic (PK) and pharmacodynamic (PD) profiles is essential. Regarded as a vital participant in host metabolism, the gut microbiota facilitates ginsenoside transformation, enhances membrane permeability, and is associated with increased systemic absorption through deglycosylation reactions mediated by bacteria [92]. Ginsenoside Rb1, a protopanaxadiol-type ginsenoside, exhibits poor oral bioavailability due to its large molecular size and hydrophilicity. Its systemic efficacy is therefore contingent upon deglycosylation by specific colonic commensals (e.g., *Bifidobacterium*, *Bacteroides*) into more hydrophobic and active metabolites such as compound K (CK) [93,94]. Studies have demonstrated this conversion both in vitro and in vivo, suggesting that microbial metabolism facilitates the subsequent absorption of CK into the systemic circulation. Similarly, Rg1, a protopanaxatriol-type saponin, undergoes microbial metabolism into bioactive derivatives such as protopanaxatriol (PPT), which exhibit potent anti-inflammatory properties [95].

This biotransformation process underscores a fundamental concept that the gut microbiota is not merely a passive target of ginsenosides but an important contributor to the pharmacological efficacy of ginsenosides, particularly for metabolites like CK that require microbial activation [96]. However, this reliance on microbial metabolism introduces a significant challenge. The metabolic capacity to generate active secondary saponins exhibits substantial inter-individual variability, suggesting that the clinical efficacy of ginsenosides is dictated by the distinct metabolic profile of the patient’s microbiome [97]. Consequently, relying solely on spontaneous in vivo biotransformation often yields unpredictable therapeutic outcomes, particularly in individuals with dysbiosis.

To optimize the efficacy of ginsenosides, recent biotechnological advancements have focused on harnessing specific probiotic strains and enzymes for precision bioconversion. Strategies involving the utilization of probiotics, such as *Lactobacillus rhamnosus* HN001, along with specialized fungal strains like *Penicillium decumbens*, are increasingly employed to ferment parent ginsenosides into rare, highly bioavailable secondary saponins [98,99]. Insight into optimizing fermentation parameters for high-yield production and establishing rigorous safety profiles provides a foundational pathway for the clinical translation of these bioactive metabolites.

### 6.2. Modulation of Gut Microbiota by Rg1 and Rb1 in IBD

Ginsenoside Rg1 has been extensively reported to modulate the gut microbiota composition and metabolic outputs in experimental IBD models. In multiple colitis models, Rg1 consistently correlated with mitigation of gut dysbiosis, characterized by enrichment of beneficial taxa such as *Lactobacillus*, *Muribaculaceae*, and *Akkermansia*, and concurrent reduction in pathogenic or pro-inflammatory bacteria such as *Escherichia-Shigella* [32]. In DSS-induced colitis, Rg1 restored microbial diversity, increased *Lachnospiraceae*, and decreased pathogenic genera such as *Staphylococcus* and *Bacteroides*, changes that were correlated with enhanced M2 macrophage polarization and inhibition of the Nogo-B/RhoA signaling pathway [27].

Beyond microbial composition, Rg1 treatment has been linked to alterations in microbiota-derived metabolites. In obese mice with ulcerative colitis, Rg1 reversed dysregulated lipid metabolism, restored α-diversity, and enriched beneficial taxa such as *Alistipes* and *Rikenellaceae*, while reducing pro-inflammatory bacteria, including *Romboutsia*, changes that paralleled correction of Th1/Th17/Th2 immune imbalance [100]. Integrated 16S rRNA sequencing and metabolomic analyses further suggest that Rg1 promotes tryptophan metabolism, increasing the levels of anti-inflammatory metabolites such as indole-3-propionic acid (IPA), indole-3-lactic acid (ILA), indole-3-carboxaldehyde (IAld), and nicotinamide [24]. These metabolites were subsequently linked to AHR signaling activation, intestinal barrier protection, and attenuated inflammation. Consistently, in 2,4,6-trinitrobenzene sulfonic acid (TNBS)-induced colitis, Rg1-derived metabolites, such as PPT, suppressed NF-κB signaling, reduced pro-inflammatory cytokines (TNF-α, IL-1β, and IL-17), and rebalanced Th17/Treg responses, alleviating colonic pathology [95]. Comparative analyses among ginsenosides further indicate that Rg1 exhibits robust anti-inflammatory activity and effectively normalizes gut metabolite profiles, particularly short-chain fatty acids (SCFA) and acylcarnitines [101].

Although no direct evidence links ginsenoside Rb1 to IBD through gut microbiota modulation, several studies provide indirect support. Administration of Rb1 has been associated with an increased abundance of *Akkermansia*, a beneficial mucin-degrading bacterium, accompanied by alterations in inflammatory mediators such as TNF-α, IL-6, and iNOS. This suggests potential immune modulation via microbiota-dependent mechanisms [102]. Furthermore, the bidirectional interaction between Rb1 and the microbiota is critical. Long-term Rb1 administration alters its pharmacokinetic profile by prolonging its half-life and reducing its absorption rate. These changes correlate with shifts in gut microbiota composition, specifically an increased abundance of *Lactobacillus* and *Bacteroides*, implicating the gut microbiota in regulating Rb1 metabolism and bioavailability [94]. Moreover, in HFD-fed mice, Rb1 administration was associated with beneficial microbial shifts, including reduced abundance of pathogenic *Helicobacter* and *Oscillospira* genera while enriching taxa such as *Adlercreutzia* and *Rikenellaceae*, which were associated with improved glucose and lipid metabolism. These metabolic improvements are hypothesized to be relevant for IBD patients with metabolic comorbidities [103]. Notably, in vitro evidence demonstrates that compound K, the major gut microbiota-metabolite of Rb1, promotes the growth of beneficial microbes such as *Akkermansia muciniphila* and *Bifidobacterium animalis*, which are known to support intestinal health and suppress inflammation [104]. In models of IBD-associated colorectal carcinogenesis, Rb1 treatment was associated with improved microbial diversity, increased SCFA-producing bacteria such as *Butyricicoccus*, and reduced pathogenic taxa, concomitant with decreased inflammatory cytokine levels [105].

Overall, Rg1 is associated with extensive alterations in gut microbiota and metabolite profiles across IBD models, though many of these findings remain correlative and require functional validation to confirm a direct microbiota-mediated mechanism. In contrast, the effects of Rb1 are largely inferred from indirect microbiota- and metabolism-related studies, highlighting a gap that requires further disease-focused investigation.

### 6.3. Potential Modulation of Gut Microbiota by Rg1 and Rb1 in MASLD and RA

Unlike IBD, where a combination of direct and indirect evidence supports microbiota-mediated effects of ginsenosides, the current literature on MASLD and RA predominantly relies on indirect evidence and extrapolations from related models. In these models, Rg1 administration has been associated with alterations in gut microbial composition, including enrichment of beneficial taxa such as *Akkermansia*, *Bifidobacterium*, and SCFA-producing members of *Ruminococcaceae* and *Lachnospiraceae*, as well as enhanced SCFA- and tryptophan-derived metabolites [106,107]. These microbial changes correlate with reduced hepatic lipid accumulation and inflammation. Based on current evidence, these effects are hypothesized to be partly mediated through regulation of macrophage M1/M2 polarization and inhibition of IL-17/NF-κB and TLR4/NF-κB signaling pathways, which are central to MASLD-related immunometabolic dysfunction [27,108,109]. Accumulating evidence has established a pivotal role for gut microbiota-derived metabolites, particularly tryptophan catabolites, in maintaining intestinal barrier integrity and modulating hepatic inflammation during MASLD development [110,111,112]. Microbial tryptophan derivatives, such as indole-3-acetic acid (IAA) and indole-3-propionic acid (IPA), activate AHR to stimulate IL-22 production, which is essential for mitigating oxidative stress and ferroptosis in the liver [113,114]. While the regulatory bioactivity of ginsenoside Rg1 on the gut microbiota has been primarily explored in colitis models, its hepatoprotective mechanisms concerning the gut-liver axis merit further investigation [27]. Recently, Wang et al. provided direct evidence that Rg1 ameliorates MASLD through gut microbiota-tryptophan metabolism-AHR/IL-22 axis in mice [49,115]. Fecal microbiota transplantation (FMT) from Rg1-treated donors upregulated intestinal and hepatic AHR/IL-22 expression and elevated 5-HIAA in recipients, supporting the conclusion that Rg1 ameliorates MASLD by promoting *Akkermansia* abundance and indole metabolite production to restore the gut–liver signaling axis.

Ginsenoside Rb1 has been associated with alterations in gut microbiota composition in MASLD-related models, like HFD-induced obesity mouse model. Specifically, Rb1 increased the abundance of *Akkermansia*, *Lactobacillus*, *Allobaculum*, and SCFA-producing members of *Ruminococcaceae* and *Lachnospiraceae*, while suppressing pro-inflammatory genera such as *Bacteroides*, *Odoribacter*, and *Turicibacter*. These microbial shifts were associated with improved intestinal barrier integrity, normalization of microbial metabolite profiles, and reduced systemic and hepatic inflammation, supporting a microbiota-mediated contribution to Rb1’s hepatoprotective effects, although direct causal validation is still required [116,117].

In the context of RA, characteristic gut dysbiosis involves a decline in SCFA-producing commensals, an increase in pro-inflammatory genera like *Bacteroides* and *Prevotella*, impaired barrier function, and altered microbial metabolites that collectively drive systemic immune activation [118,119,120]. This dysbiosis profile shares notable similarities with the microbial shifts observed following Rg1 treatment. Specifically, the observation that Rg1 treatment was associated with restoration of SCFA producers suggests that its microbiota-regulating properties could be therapeutically relevant in RA. Although direct evidence in RA remains limited, the observation that Rb1 alters the gut microbiota suggests it may also modulate immune dysregulation and inflammation in RA through analogous pathways.

## 7. Discussion

Ginseng, a traditional herbal medicine widely used in East Asia, exhibits broad pharmacological activities, including anti-inflammatory, antioxidative, and immunoregulatory effects. Its major bioactive constituents, ginsenosides, have attracted increasing scientific attention for their ability to modulate multiple signaling networks involved in chronic immune-metabolic disorders [18].

**Figure 4:**
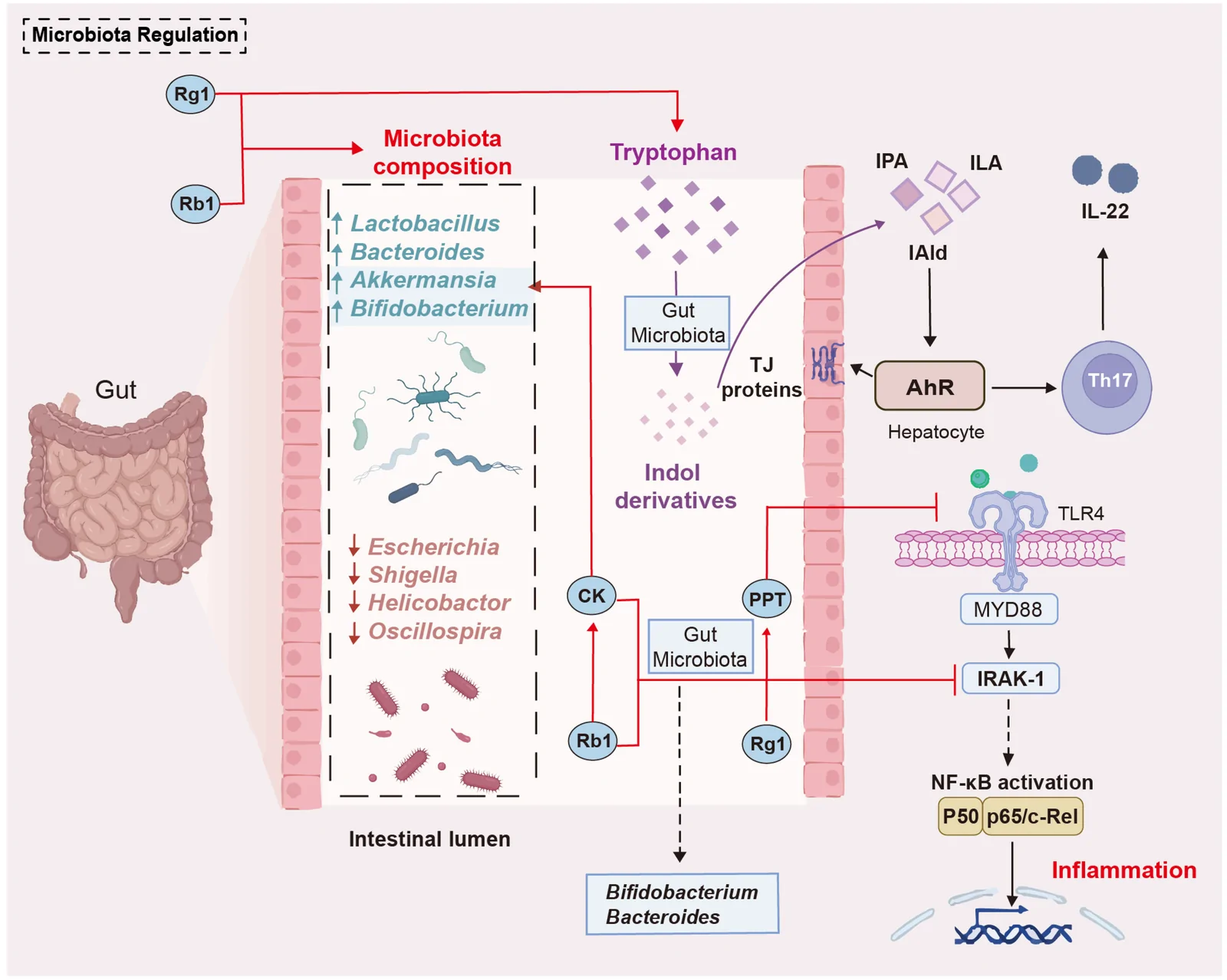
Ginsenosides Rg1 and Rb1 regulate intestinal microbiota by increasing beneficial taxa and decreasing potential pathogens. These modifications promote tryptophan metabolism and generation of indole derivatives by gut microbiota. The metabolites activate AHR signaling in hepatocytes, influencing the Th17/IL-22 axis and tight junction proteins, while inhibiting the TLR4/MYD88/IRAK-1/NF-κB inflammatory pathway. (Generated with Adobe Illustrator v29.5.1).

**Figure 5:**
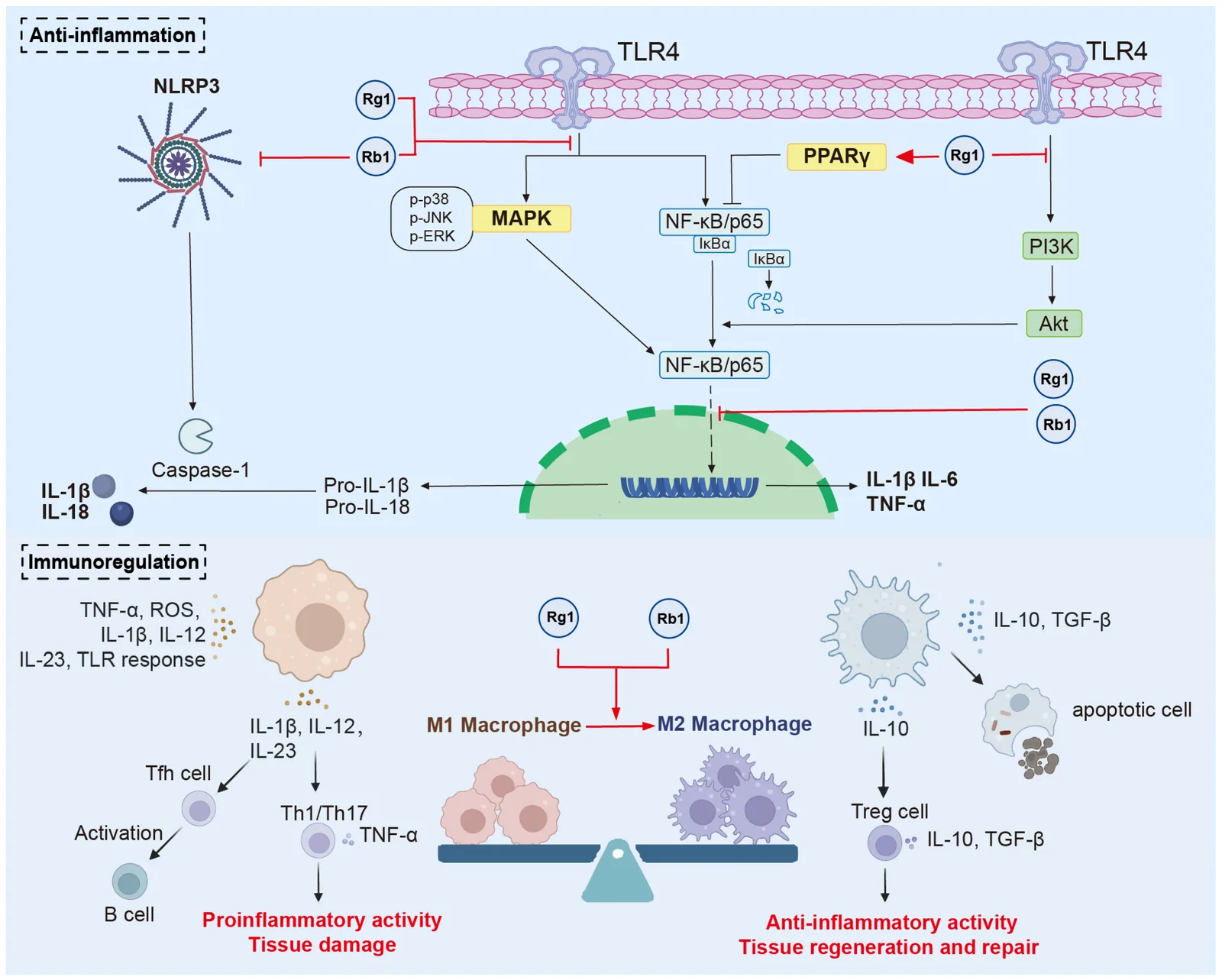
Ginsenosides Rg1 and Rb1 suppress TLR4/MAPK and NF-κB activation, reducing pro-inflammatory cytokines (TNF-α, IL-1β, IL-6) and NLRP3 inflammasome-mediated IL-1β/IL-18 release. Rg1 and Rb1 facilitate M1-to-M2 macrophage transition, shifting the microenvironment from proinflammatory tissue damage to anti-inflammatory repair. Additionally, they modulate T-cell responses by inhibiting Th1/Th17 activation and promoting Treg cell differentiation (IL-10, TGF-β), thereby enhancing tissue regeneration. PPARγ and PI3K/Akt pathways are also involved in these protective mechanisms. (Generated with Adobe Illustrator v29.5.1).

**Figure 6:**
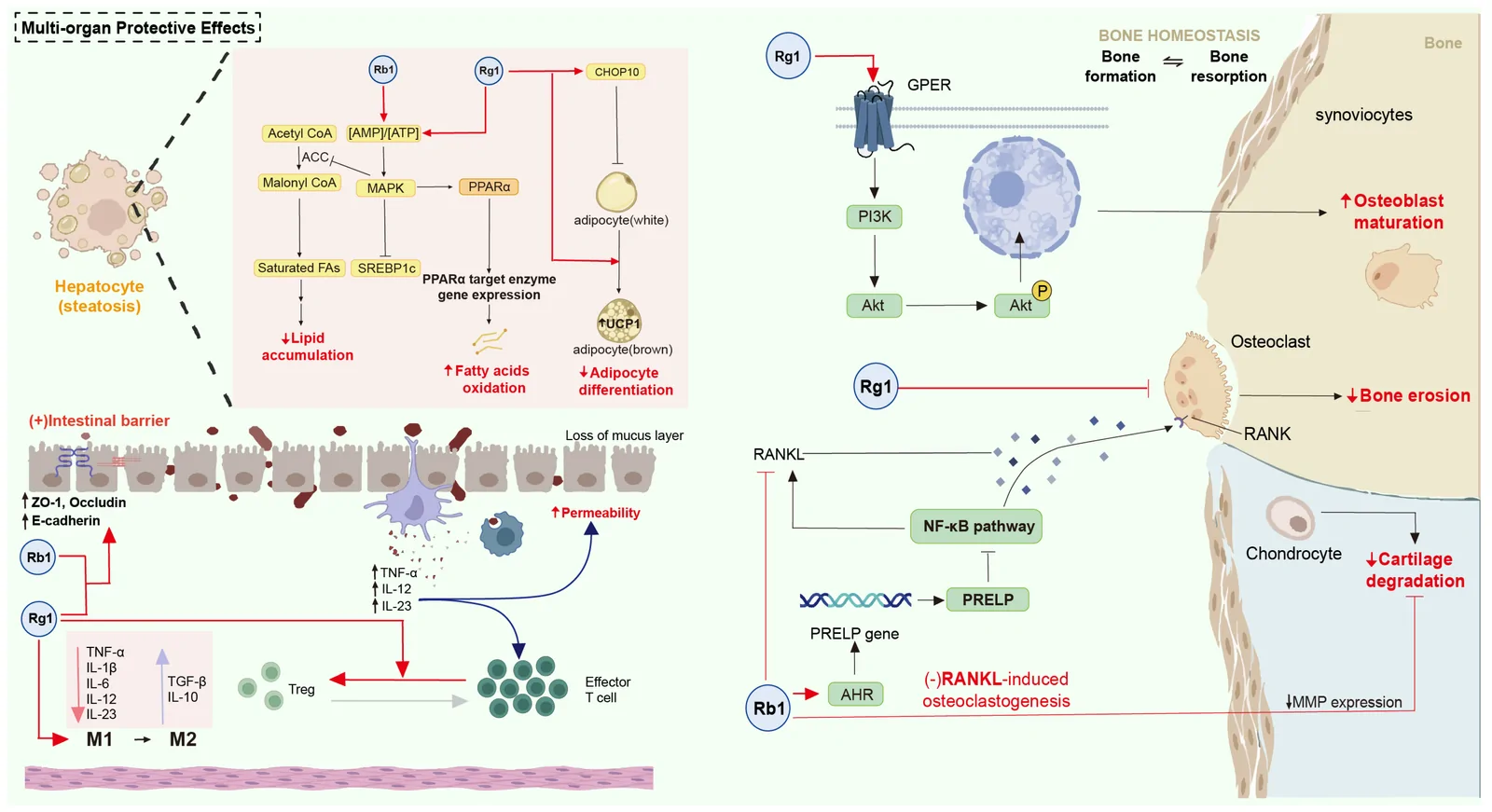
Ginsenosides Rg1 and Rb1 maintain intestinal barrier integrity, protect against hepatic steatosis by regulating lipid metabolism, and regulate bone homeostasis by inhibiting RANKL-induced osteoclastogenesis and the NF-κB pathway. In the intestine, Rg1 and Rb1 preserve barrier proteins (ZO-1, Occludin, E-cadherin), reduce permeability, and facilitate M1-to-M2 macrophage polarization. In hepatocytes, Rg1 inhibits adipocyte differentiation, while both Rg1 and Rb1 modulate AMPK/PPARα and GSK3β/ACC pathways to promote fatty acid oxidation and suppress lipid accumulation. In bone, Rg1 activates GPER/PI3K/Akt signaling to promote osteoblast maturation and inhibit bone erosion, whereas Rb1 suppresses RANKL-induced osteoclastogenesis via the NF-κB/PRELP and AhR pathways, reducing cartilage degradation and MMP expression. (Generated with Adobe Illustrator v29.5.1).

Our analysis reveals that despite their distinct clinical manifestations, these conditions share a convergent molecular etiology characterized by “barrier-immunity-metabolism” dysfunction. The therapeutic efficacy of Rg1 and Rb1 is not attributable to a single pathway but arises from their capacity to simultaneously modulate immunometabolic networks. Specifically, Rg1 and Rb1 remodel the gut microbiota by enriching beneficial taxa and reducing potentially pro-inflammatory taxa, thereby promoting tryptophan metabolism and downstream AHR signaling; this enhances tight junction integrity and suppresses the TLR4/MyD88/IRAK-1/NF-κB axis (Figure 4). In parallel, both compounds exert direct anti-inflammatory and immunoregulatory effects, suppressing TLR4/MAPK and NF-κB activation, inhibiting NLRP3 inflammasome-mediated cytokine release, facilitating M1-to-M2 macrophage polarization, and promoting Treg differentiation through PPARγ and PI3K/Akt signaling (Figure 5). These actions converge on multi-organ protection: Rg1 and Rb1 preserve intestinal barrier proteins, regulate hepatic lipid metabolism via the AMPK/PPARα axis, and maintain bone homeostasis, with Rg1 promoting osteoblast maturation through GPER/PI3K/Akt signaling and Rb1 suppressing RANKL-induced osteoclastogenesis (Figure 6).

This multi-target strategy offers a distinct advantage over conventional therapies, potentially ameliorating oxidative stress, immune hyperactivation, and metabolic dysfunction.

Network pharmacology analysis predicted a high degree of target overlap among IBD, MASLD, and RA, prioritizing *TNF*, *NFKB1*, *PPARG*, and *MAPK1* as putative hubs. The high centrality of these nodes in the PPI network reflects their general importance in immunometabolic disorders. However, network topology alone does not establish them as direct or specific targets of Rg1 or Rb1. Critically, only a subset of these predicted hubs has direct biochemical binding or biochemical support. For instance, Rb1 has been experimentally demonstrated to bind directly to TLR4 and block its dimerization, thereby suppressing downstream NF-κB and MAPK signaling cascades [37]. Similarly, Rg1 can activate PPARγ [74]. For *NFKB1* and *MAPK1*, the available evidence is restricted to pathway-level modulation rather than direct target engagement. Specifically, Rb1 suppresses IκBα phosphorylation and downstream NF-κB activation, Rb1 modulates TLR4-mediated NF-κB and MAPK signaling, and Rg1 attenuates p38 MAPK/NLRP3 signaling [25,31,37,62]. These findings confirm that the corresponding pathways are functionally modulated, but they do not demonstrate direct binding of Rg1 or Rb1 to NF-κB or MAPK1 proteins. Notably, although both ginsenosides reduce TNF-α levels across multiple disease models, direct binding to TNF-α has not been demonstrated, and its placement as a hub should be regarded as a network prediction rather than a validated interaction.

Given their central roles across IBD, MASLD and RA, we further delineate their biological function. TNF-α and NF-κB act as central amplifiers of the inflammatory cascade. The pro-inflammatory cytokine TNF-α activates downstream NF-κB and MAPK pathways, which in turn promote secretion of IL-1β and IL-6. Persistent activation of this axis drives epithelial inflammation, hepatic injury, impaired insulin signaling, and lipid accumulation, thereby linking immune activation to metabolic dysregulation [121]. As a major downstream effector of TNF-α signaling, NF-κB regulates a broad spectrum of genes involved in immunity, inflammation and cell survival. Sustained NF-κB activation amplifies immune responses in the gut, liver and joints, and contributes to immune-mediated disease pathogenesis by promoting cytokine production, immune cell infiltration and tissue damage [122]. In contrast to these pro-inflammatory drivers, PPAR-γ, encoded by *PPARG*, is a nuclear receptor and transcription factor coordinating metabolic and inflammatory cues. Its activation suppresses NF-κB-mediated cytokine production while simultaneously enhancing glucose and lipid metabolism to promote metabolic homeostasis. In experimental models of colitis and other inflammatory conditions, PPAR-γ activation consistently reduces pro-inflammatory mediator expression and alleviates tissue injury [123,124], demonstrating its pivotal role in restoring immunometabolic homeostasis. Furthermore, cellular responses to inflammatory and metabolic stress are shaped by kinases such as MAPK. As mitogen-activated protein kinases, the MAPK family (comprising ERK, JNK and p38 cascades) transduces extracellular stress signals into coordinated changes in gene expression, cytokine production and cell fate, processes essential for cellular adaptation under inflammatory or metabolic stress [31,37].

A pivotal insight from our study is the identification of the gut microbiota as a potential contributor to ginsenoside efficacy, though the directionality and causal nature of this relationship remain to be established. To elucidate the specific microbial-host interactions underlying the effects of Rg1 and Rb1, we performed a gut microbiota–host gene association analysis across IBD, MASLD, and RA. This analysis identified a core set of conserved microbial taxa that align with and extend current evidence.

For Rg1, *Streptococcus salivarius* and *Lacticaseibacillus paracasei* were recurrently identified across all three disease networks, whereas *Lactiplantibacillus plantarum* appeared in the IBD and MASLD networks and *Faecalibacterium prausnitzii* in the IBD and RA networks, pointing to a partially shared microbial-regulatory framework. Notably, the association of the anti-inflammatory commensal *Faecalibacterium prausnitzii* with Rg1 in IBD and RA networks is consistent with experimental reports demonstrating that Rg1 treatment is associated with alterations in beneficial taxa, including *Lactobacillus*, *Muribaculaceae*, and *Akkermansia*, while being correlated with reductions in pro-inflammatory bacteria such as *Escherichia-Shigella* and *Bacteroides* [27,32,125]. However, these 16S rRNA sequencing-based findings reflect correlations, and causal relationships require further validation via FMT or in vitro co-culture.

Similarly, the Rb1-associated network highlights a distinct microbial profile centered on *Akkermansia muciniphila*, *Escherichia coli*, and *Parabacteroides goldsteinii*. The repeated association between Rb1 and *Akkermansia muciniphila* in network models is consistent with experimental observations that Rb1 administration is correlated with shifts in this mucin-degrading bacterium, which is involved in intestinal barrier function and metabolic homeostasis [126]. Direct experimental evidence showed that Rb1 intervention in rats significantly increased the relative abundance of *Bacteroides cellulosilyticus* and specific glycoside hydrolase families (GH2, GH20), and these microbial changes were robustly associated with altered pharmacokinetics of Rb1 [94,102].

Crucially, our analysis suggests that these microbial shifts may translate into host regulation through a metabolic bridge, representing a putative intermediary mechanism that remains to be experimentally validated. The identification of unique metabolites produced by these key strains (ranging from 5 to 16 for Rg1 and 20 to 30 for Rb1 across the three disease networks) supports the potential role of the microbiota as a metabolic intermediary. However, this proposed therapeutic trajectory is largely derived from in silico predictions and correlative evidence, and in vivo alterations of these metabolites following ginsenoside intervention remain unvalidated in the present study. Mechanistically, this pathway is likely initiated by ginsenoside-mediated selective modulation of the gut microbiota, which reshapes the intestinal metabolome toward the enrichment of bioactive mediators, including tryptophan derivatives, short-chain fatty acids (SCFAs), and the secondary ginsenoside metabolite CK [92]. These metabolites are predicted to interact with host targets, specifically activating PPARγ to regulate metabolism, NF-κB to inhibit inflammation, and MAPK to modulate immune signaling. This multi-step pathway implies that the anti-inflammatory and metabolic effects of Rg1 and Rb1 are substantially amplified or modulated by the gut microbiota, but the precise causal contributions of individual bacterial taxa and metabolites remain to be fully elucidated. Definitive causal validation will require interventional experiments such as fecal microbiota transplantation, gnotobiotic colonization, metabolite supplementation, or in vitro co-culture assays. By converting parent ginsenosides into bioactive derivatives or stimulating the production of endogenous microbial metabolites, the microbiota functions as a critical transducer that converts oral ginsenoside intake into systemic regulation of host gene networks.

Future studies should prioritize the experimental validation of these specific ginsenoside-microbiota-metabolite-host interactions, particularly in MASLD and RA, where direct causal evidence remains less extensive than in IBD. These findings are summarised according to their evidence level in Supplementary Table S2.

Although anti-TNF therapy has transformed IBD management, primary non-response occurs in approximately 10–30% of patients, and 23–46% lose response over time [127]. Therapies targeting IL-12/23 axis offer an alternative mechanism, yet a subset of patients experience a delayed onset of effect. Moreover, the use of JAK inhibitors is constrained by safety concerns regarding potential risks such as thrombosis or infection [128]. Beyond immune modulation, two other critical therapeutic gaps persist. Effective restoration of damaged epithelial barrier integrity remains an elusive goal, and microbiota-based interventions show variable efficacy due to disease heterogeneity. These collective limitations highlight a pressing clinical need for multi-target approaches capable of simultaneously addressing the interconnected facets of IBD pathogenesis, with ginseng representing a promising drug candidate worthy of further investigation. In IBD, current registered clinical trials predominantly focus on UC and utilize complex ginseng formulations such as Qingshen decoction (ITMCTR2025001310) and Sanrenkushen Formula (ITMCTR2024000870), rather than purified Rg1 or Rb1, often combined with standard therapy (Table 4) [129,130]. The scale of these investigations varies, with sample sizes ranging from 60 to 180 participants, and trials are currently at various stages. This clinical research landscape underscores the translational interest in harnessing the pleiotropic potential of ginseng constituents while simultaneously indicating a gap in clinical evidence specifically dedicated to well-defined, single ginsenoside compounds.

In spite of intense research efforts, effective pharmacological treatments for MASLD continue to be scarce. No currently approved therapy comprehensively addresses all pathogenic axes of the disease. Many agents focus narrowly on single aspects, such as lipid lowering, insulin sensitization, or anti-inflammation, yet none fully resolve the concurrent metabolic and immune dysfunction. This therapeutic gap has motivated investigation into multi-target natural compounds like ginsenosides. For MASLD, clinical trials primarily assess complex preparations such as Korean red ginseng, ginseng oligopeptide preparations, or ginseng extracts (Table 4) [131,132]. These studies typically enroll patients with conditions underpinning MASLD, such as metabolic syndrome. Most are either completed or in progress, reflecting ongoing interest in ginseng-derived metabolic and hepatic benefits, but their multi-component nature limits what can be inferred about the efficacy of purified Rg1 or Rb1.

While JAK inhibitors and biologics are effective for RA, they are associated with long-term risks of infection and thrombosis, and they often fail to resolve the metabolic comorbidities that drive cardiovascular risk. In RA, evidence is still sparse, with limited studies on Wisconsin ginseng and ginsenoside compound K targeting fatigue and disease activity (ITMCTR1900002626, NCT02260336) (Table 4) [133,134]. Of particular note is the absence of phase II/III trials specifically evaluating single ginsenosides (Rg1 or Rb1). This gap between the mechanistic evidence from preclinical models and the lack of precise clinical data represents a major hurdle for clinical translation.

To bridge the translational gap and enhance clinical utility, recent research has prioritized several strategic innovations for ginsenoside delivery and application. Nanoparticle-based delivery systems have been engineered to overcome the limitations of poor stability, low bioavailability, and lack of tissue specificity. Among these, chitosan-based micro- and nano-encapsulation has emerged as a versatile strategy, offering protection against degradation, controlled release, and site-specific delivery [135]. For instance, glycogen-based pH- and redox-sensitive nanoparticles loaded with ginsenoside Rh2 demonstrated significant efficacy in experimental colitis by rapid release from inflammatory sites [136]. Similarly, copper-luteolin nanocomplexes demonstrate multifaceted modulation of intestinal inflammation and microbial homeostasis [25,137]. Other platforms currently under investigation include PLGA nanoparticles, liposomes, exosome-based carriers, and hydrogel-based colon-targeted formulations, all designed to optimize localized delivery to inflamed intestinal tissue while minimizing systemic exposure and off-target effects. Furthermore, structural modifications of ginsenosides via enzymatic conversion or glycosylation have proven effective in improving solubility, metabolic stability, and receptor affinity. When combined with advanced delivery matrices, these modified derivatives show superior anti-inflammatory and immunomodulatory potency in preclinical models of metabolic-inflammatory disorders [25,137]. Finally, multi-target combinatorial strategies are increasingly recognized as essential to exploit the full therapeutic potential of ginsenosides. Co-administration with probiotics, FXR agonists, or small-molecule immunomodulators, such as JAK inhibitors, offers a synergistic approach. Preclinical evidence suggests these combinations synergistically recalibrate microbial communities, restore barrier function, and simultaneously suppress pathogenic immune signaling across the gut-liver-joint axis. Such integrative modalities address the multi-organ pathophysiology underlying IBD, MASLD, and RA by targeting overlapping inflammatory and metabolic pathways.

It is noteworthy that ginsenosides Rg1 and Rb1 are characterized by limited oral bioavailability (Rb1: 1.18–4.35%; Rg1: 6.06–18.40% in rodents) and undergo extensive biotransformation by gut microbiota into metabolites such as compound K (from Rb1) and Rh1 (from Rg1) [138,139]. The relatively high oral doses employed in the studies listed in Table 1, Table 2 and Table 3 are within the range commonly used in preclinical ginsenoside research to compensate for low oral bioavailability and to ensure adequate exposure of both parent compounds and active microbial metabolites at target sites. Direct plasma or tissue concentration data were not consistently reported in the primary studies summarized in Table 1, Table 2 and Table 3. However, the selected dose ranges are consistent with those used in ginsenoside pharmacokinetic studies in which oral administration of Rg1 (50–200 mg/kg) or Rb1 (~10–100 mg/kg) resulted in detectable levels of parent compounds and/or bioactive metabolites in plasma, liver, and intestinal tissues [140]. Nevertheless, it should be noted that these preclinical pharmacokinetic data were largely obtained in healthy rodents. Direct translation to human therapeutic exposures remains uncertain due to interspecies differences in metabolism, gut microbiota composition, and disease-state pathophysiology.

However, translation to clinical application remains limited. Most studies are confined to small animal models, and multi-organ interactions have yet to be fully characterized in large-animal or human systems. Rigorous clinical evaluation of safety, pharmacokinetics, and efficacy is required to validate the potential of ginsenoside-based interventions in metabolic-inflammatory diseases. In studies examining ginseng and gut microbiota, detection methods have largely been confined to 16S rDNA sequencing, failing to adequately and specifically elucidate microbial alterations. Future trials should move beyond simple phenotypic endpoints and incorporate multi-omics biomarkers (e.g., serum metabolomics, fecal microbiome sequencing).

## 8. Limitations

Several limitations of this study should be acknowledged. First, the network pharmacology analysis is inherently subject to database dependency and prediction bias. Target identification relied on publicly available databases (e.g., OMIM, GeneCards, TTD), whose coverage, annotation standards, and update frequencies vary, potentially introducing selection bias toward well-characterized genes. Drug target prediction was primarily based on chemical structural similarity or reverse pharmacophore matching. These computational methods cannot fully simulate complex in vivo pharmacokinetic processes or dynamic protein conformational changes. Thus, predicted targets may deviate from actual binding conditions in biological systems. Furthermore, the construction of the PPI network involved arbitrary thresholds for interaction confidence scores and network topology parameters. These included the relevance score cutoff for GeneCards, the minimum required interaction score in STRING, and the selection of top 30 targets based on centrality measures. While these thresholds were guided by existing research experience, they inherently possess a degree of subjectivity and may influence the composition of identified hub targets. Therefore, the results should be interpreted with caution. Importantly, with the exception of TLR4, which Rb1 has been experimentally shown to bind directly [37], and PPARγ, for which functional activation by Rg1 has been demonstrated [74], the predicted interactions between ginsenosides and hub nodes (TNF, NFKB1, MAPK1) rest primarily on pathway-level evidence and lack direct biochemical validation, such as binding assays, enzymatic assays, or genetic loss-of-function confirmation.

Second, preclinical studies in Table 1, Table 2 and Table 3 are heterogeneous in animal species, disease models, dosing regimens, and outcome measures, limiting cross-study comparability. Additionally, the low oral bioavailability of Rg1 and Rb1 and their extensive gut microbial biotransformation challenge the extrapolation of preclinical doses to human exposures, as discussed above.

Finally, clinical evidence remains limited. Most registered trials evaluated complex ginseng formulations rather than purified Rg1 or Rb1, and phase II/III trials for single ginsenosides are lacking. Rigorous clinical evaluation of safety, pharmacokinetics, and efficacy is needed to substantiate their translational potential.

## 9. Conclusions and Prospect

To date, ginsenosides Rg1 and Rb1 are recognized as pivotal bioactive components, particularly for complex inflammatory and metabolic disorders. Despite the distinct clinical presentations of IBD, MASLD, and RA, the convergence of these conditions on a common “barrier-immunity-metabolism” dysfunction remains a significant insight. This review comprehensively explains the shared therapeutic mechanisms of Rg1 and Rb1, integrating recent research progress with insights from network pharmacology and host-microbe association analyses. Specifically, we highlight the roles of Rg1 and Rb1 in co-modulating the gut microbiota and host signaling hubs, particularly TNF, NF-κB, and PPAR-γ, though the causal relationship between these microbial and host factors requires further experimental validation. However, the majority of current research primarily highlights their pleiotropic effects, while the exact molecular targets and pharmacological pathways remain largely undefined. Consequently, precise molecular interactions require further validation through multi-omics integration and high-throughput screening. Moreover, the clinical utility of parent ginsenosides is currently deemed suboptimal due to their low bioavailability and reliance on variable microbial biotransformation. Thus, future research should be directed towards recent innovations in precision delivery systems, including nanoparticle formulations, liposomes, and polymeric micelles, which have shown great promise. In conclusion, these advancements provide a solid foundation for the clinical application of Rg1 and Rb1, paving the way for an alternative to conventional immunosuppressive therapies.

## Figures and Tables

**Table 1:** Mechanisms of ginsenosides Rg1 and Rb1 in IBD: evidence from DSS and TNBS models.

Ginsenosides	Animal or Cell Line	Model	IBD Relevance	Sample Size	Treatment Dosage	Treatment Duration	Effects	Pathway	Ref.
Rg1	BALB/c mice	DSS	UC	*n* = 10/group	50, 100, 200 mg/kg	7 days	↓ TNF-α, IFN-γ	NF-κB pathway	[24]
							↑ IL-4, ZO-1		
Rg1	C57BL/6 mice	DSS	UC	*n* = 10–15/group	20 mg/kg	14 days	↓ IL-6, IL-33, TNF-α, IL-1β, and IL-17A, Th17	TLR4/MyD88-dependent pathway	[25]
							↑ IL-10, Treg		
Rg1	BALB/c mice	DSS	UC	*n* = 10/group	200 mg/kg	10 days	↓ IL-6, TNF-α, Tfh9, Tfh17, PD-1^+^Treg, PD-L1^+^Treg	PI3K/Akt pathway	[26]
							↑ IL-10, Tfr, Treg		
Rg1	BALB/c mice	DSS	UC	*n* = 8/group	200 mg/kg	14 days	↓ IL-6, IL-33, CCL-2, TNF-α, CD11b^+^F4/80^+^TLR4^+^, iNOS^+^ M1 macrophages	Nogo-B–RhoA–ROCK1 pathway	[27]
							↑ IL-4, IL-10, CD206^+^, CD163^+^ M2 macrophages		
Rb1	C57BL/6 mice	DSS	UC	*n* = 6/group	20, 40, 80 mg/kg	10 days	↓ TNF-α, IL-6	PPARγ/NF-κB pathway	[28]
							↑ IL-10, ZO-1, Occludin, E-cadherin		
Rb1	C57BL/6 mice	DSS and TNBS	UC and CD	*n* = 6/group	20, 40 mg/kg	7 days	↓ GRP78, PERK, CHOP, caspase-12	Hrd1 pathway	[29]
Rb1	ICR mice	TNBS	CD	*n* = 7/group	10, 20 mg/kg	3 days	↓ TNF-α, IL-1β, IL-6, COX-2, iNOS	NF-κB, MAPKs pathway	[30]
							↑ IL-10		

↑, increased; ↓, decreased.

**Table 2:** Mechanisms of ginsenosides Rg1 and Rb1 in MASLD: evidence from MASLD-relevant models.

Ginsenosides	Animal or Cell Line	Model	MASLD Relevance	Sample Size	Treatment Dosage	Treatment Duration	Effects	Pathway	Ref.
Rg1	SD rats	HSHF diet	Diet-related MASLD	*n* = 10/group	30, 60 mg/kg	4, 8 weeks	↓ GLU, INS, TG, TC, IL-1, IL-6, IL-8, IL-18, TNF-α	PPAR-α pathway	[44]
Rg1	SD rats	HSHF diet	Diet-related MASLD	*n* = 10/group	10, 25, 50 mg/kg	4 weeks	↓ ALT, AST, ALP, TC, TG, LDL, IL-1β, IL-6, TNF-α, NF-κB, G6Pase	PI3K/Akt pathway	[48]
							↑ HDL, p-Akt		
Rg1	C57BL/6J mice	CDAHFD	Fibrogenic mechanisms in advanced MASLD	*n* = 10/group	60, 120 mg/kg	4 weeks	↓ liver inflammation and hepatic lipid deposition	gut microbiota-tryptophan metabolism-AHR/IL-22 pathway	[49]
							↑ 5-hydroxyindoleacetic acid		
Rg1	HepG2 cells	Palmitic acid	Hepatocellular injury mechanisms	-	0, 10, 20, 40, 80, 100 μg/mL	6, 24 h	↓ lipid droplets and intracellular TG, ALT, AST, IL-1β, IL-6, TNF-α	AMPKs, NF-κB pathway	[45]
							↑ p-AMPK, p-ACCα		
Rg1	C57BL/6 mice	D-galactose	Oxidative stress–associated mechanisms relevant to MASLD progression	*n* = 10/group	40 mg/kg	27, 42 days	↓ SA-β-gal, p53, p21, ALT, AST, IL-1β, IL-6, MCP-1, MDA	FOXO1 pathway	[50]
							↑ FOXO1 protein, SOD, CAT		
Rg1	HHl-5 cells	medium- and long-chain fat emulsion	Lipid handling and overload responses	-	0.2, 0.4, 0.6 mM	20, 24 h	↓ lipid accumulation, apoptosis, Bax, SGPL1	sphingosine signaling pathway	[51]
							↑ Bcl-2, p-Akt, p-ERK1/2		
							↑ fatty acid oxidation, CPT1, AMP/ATP ratio		
Rb1	C57BL/J mice	HFD	Metabolic dysfunction in early MASLD	*n* = 8/group	10 mg/kg	8 weeks	↓ hepatocyte apoptosis, cytoplasmic HMGB1	PPAR-γ pathway	[52]
							↑ PPAR-γ		
Rb1	C57BL/6J mice	HFD	Metabolic dysfunction in early MASLD	*n* = 5/group	40 mg/kg	14 days	↓ lipid accumulation	miR-128/TFEB pathway	[53]
							↑ autophagic flu, TFEB nuclear translocation		

↑, increased; ↓, decreased.

**Table 3:** Mechanisms of ginsenosides Rg1 and Rb1 in RA: evidence from established RA models and related bone, cartilage, and inflammatory models.

Ginsenosides	Animal or Cell Line	Model	RA Relevance	Sample Size	Treatment Dosage	Treatment Duration	Effects	Pathway	Ref.
Rg1	DBA/1J mice	CIA	Classic autoimmune RA model	*n* = 8/group	12.5 mg/kg	14 days	↓ Arthritis score, Cartilage destruction, TRAP^+^/Cathepsin K^+^ OCs	MAPKs, NF-κB pathway	[71]
Rg1	DBA/1J mice	CIA	Classic autoimmune RA model	*n* = 6/group	15 mg/kg	28 days	↓ CD4^+^ T cells, M1 macrophages, TNF-α, IL-6	Not specified	[72]
							↑ CD8^+^ T cells		
Rg1	TNF-Tg mice	TNF-Tg	TNF-α–driven RA pathology	*n* = 6/group	20 mg/ kg	12 weeks	↓ IL-6, TNF-α	Not specified	[73]
Rg1	SD rats	AIA	Cellular immunity in RA	*n* = 3/group	5, 10, 20 mg/kg	14 days	↓ Paw swelling, arthritis index, Joint inflammation, cartilage damage, TNF-α, IL-6,	PPAR-γ/NF-κB pathway	[74]
Rg1	AB strain zebrafish	GIOP	RA-associated osteoporosis	*n* = 20/group	12.5, 25, 50, 100, 200, 400 μM	5 days	↑ Vertebral mineralization, runx2a, osteocalcin, alp mRNA	PI3K/Akt pathway	[75]
Rg1	RAW264.7 cells	RANKL	Bone erosion in RA		1, 10, and 100 μg/mL	24, 48, 72, 96 h	↓ TRAP, Trap, CTSK, CTR, MMP-9 mRNA, c-Fos, c-Jun, NFATc1	MAPKs, NF-κB pathway	[71]
Rg1	MC3T3-E1 cells and BMSCs	GIOP	RA-associated osteoporosis	-	12.5, 25, 50, 100, 200, 400 μM	24, 48 h; 7,10, 14 days	↑ ALP, Runx2a/OCN/ALP mRNA	PI3K/Akt pathway	[75]
Rg1	RAW264.7 cells	LPS	Innate inflammatory responses		12.5, 25, and 50 μM	24 h	↓ TNF-α, IL-6	PPAR-γ/NF-κB pathway	[74]
							↑ PPAR-γ		
Rb1	DBA/1J mice	CIA	Classic autoimmune RA model	*n* = 10/group	1.25, 2.5mg/kg	18 days	↓ TNF-α	TNF-α signaling pathway	[76]
Rb1	SD rats	ACLT + MMx	Joint destruction mechanisms	*n* = 5/group	300mM	4 weeks	↓ IL-1β	Not specified	[77]
Rb1	SD rats	DEX	Therapeutic modulation in RA	*n* = 6/group	3, 6 mg/kg	12 weeks	↓ Tb.Sp, NF-κB p65	AHR–PRELP–NF-κB pathway	[78]
							↑ BMD, BV/TV, Tb.N, Tb.Th		
Rb1	SD rats	ACLT	Joint destruction mechanisms	*n* = 10/group	80 mM	6 weeks	↓ MMP-13, Notch1, JAG1	Notch pathway	[79]
							↑ CII		
Rb1	Lewis rats	R97-116 peptide	Autoantigen recognition in RA	*n* = 7/group	2.6, 3.7 mg/kg	37 days	↓ IL-17^+^ cells, IgG1/IgG2a antibodies	Not specified	[80]
							↑ Treg, IL-10		
Rb1	Raw264.7 cells	RANKL	Bone erosion in RA	-	10 μM	1, 4 days	↓ TNF-α mRNA, c-Fos, NFATc1, JNK/p38 phosphorylation, NF-κB	NF-κB, JNK / p38 MAPK pathway	[81]
Rb1	Rat primary osteoblasts	DEX	Therapeutic modulation in RA	-	0.0145 mg/mL	14 days	↓ NF-κB p65	AHR–PRELP–NF-κB pathway	[78]
							↑ ALP, Runx2, OPN, OCN		
Rb1	Chondrocytes, fibroblast-like synoviocytes	LPS, IL-1β	Innate inflammatory responses, IL-1β–mediated tissue damage	-	0.005, 0.01, 0.015, 0.03, 0.05, 0.125, 0.25, 0.5, 1, 2 mg/mL	16 h	↓ TNF-α	TNF-α signaling pathway	[76]
Rb1	C5.18 rat chondrocytes	IL-1β	IL-1β–mediated tissue damage	-	100 μg/mL	24 h	↓ MMP13 mRNA, Col X expression	Not specified	[77]
Rb1	SW 1353 chondrosarcoma cells	IL-1β	IL-1β–mediated tissue damage	-	20, 40, 80 μM	24 h	↓ MMP-13, Notch1, JAG1	Notch pathway	[79]
							↑CII		

↑, increased; ↓, decreased.

**Table 4:** Registered clinical trials of ginseng-containing preparations that contain Rg1/Rb1 among multiple constituents, identified from ClinicalTrials.gov (https://clinicaltrials.gov), WHO International Clinical Trials Registry Platform (https://trialsearch.who.int), and ITMCTR (http://itmctr.ccebtcm.org.cn) (accessed on 6 September 2026).

Conditions	Intervention	Drugs	Constituent Ginsenosides (Rg1, Rb1)	Phase	Identifier
Metabolic Syndrome	Whole ginseng powder	White Korean Ginseng	Rg1, Rb1	Phase 1	NCT01699074 (C)
Metabolic Syndrome	Whole ginseng powder	Korean red ginseng	Rb1, Rg1	N/A	NCT00976274 (C)
Metabolic Syndrome	Whole ginseng powder	Korean red ginseng	Rb1, Rg1	N/A	NCT02034136 (N)
Metabolic Syndrome	Whole ginseng powder	Red ginseng	Rb1, Rg1	N/A	KCT0004823 (A)
UC	TCM formula	Qingshen Decoction	Rb1	Pilot clinical trial	ITMCTR2025001310 (R)
UC	TCM formula	Sanren Kushen Decotion	Rb1	Pilot clinical trial	ITMCTR2025000153 (N)
UC	TCM formula	Sanrenkushen Formula	Rb1	Pilot clinical trial	ITMCTR2024000870 (N)
UC	TCM formula	Shenqing capsule	Rb1	Phase 0	ITMCTR2000003683 (N)
UC	TCM formula	Qingshenyin	Rb1	Pilot clinical trial	ITMCTR2026000535 (N)
Liver Dysfunction	Whole ginseng powder	Korean red ginseng	Rb1, Rg1	N/A	NCT03945123 (C)
Liver Dysfunction	Standardized extract	White ginseng extract	Rg1, Rb1	N/A	KCT0004080 (C)
Liver Dysfunction	Standardized extract	Panax Ginseng C.A. Mey Extract	Rg1, Rb1	N/A	NCT03775837 (R)
MASLD	Whole ginseng powder	Korea Red ginseng	Rb1, Rg1	Phase 4	NCT02331589 (C)
MASLD	Whole ginseng powder	GBCK25	Rg1	N/A	NCT03260543 (C)
MASLD	Standardized extract	Ginseng oligopeptide (Ginseng extract)	Rb1, Rg1	N/A	NCT06167902 (N)
RA	Whole ginseng powder	Wisconsin Ginseng (Panax quinquefolius)	Rg1, Rb1	Phase 2	ITMCTR1900002626 (N)
RA	Whole ginseng powder	Panax Notoginseng Powder	Rb1	N/A	NCT02260336 (R)

Trial status: A, active but not recruiting; R, recruiting; C, completed; N, Not yet recruiting.

## Data Availability

No new data were created or analyzed in this study. Data sharing is not applicable to this article.

## Supplementary Materials

The following supporting information can be downloaded at: https://www.mdpi.com/article/10.3390/microorganisms14102246/s1, Figure S1. Protein–protein interaction (PPI) network of the targets shared between ginsenoside Rg1 and IBD. Node size and color intensity are proportional to the degree value of each protein. (Cytoscape); Figure S2. Protein–protein interaction network of the targets shared between ginsenoside Rg1 and IBD, as exported from STRING. Edge colors denote the type of evidence supporting each interaction. (STRING); Figure S3. PPI network of the targets shared between ginsenoside Rb1 and IBD. Node size and color intensity are proportional to the degree value of each protein. (Cytoscape); Figure S4. Protein–protein interaction network of the targets shared between ginsenoside Rb1 and IBD, as exported from STRING. Edge colors denote the type of evidence supporting each interaction. (STRING); Figure S5. PPI network of the targets shared between ginsenoside Rg1 and MASLD. Node size and color intensity are proportional to the degree value of each protein. (Cytoscape); Figure S6. Protein–protein interaction network of the targets shared between ginsenoside Rg1 and MASLD, as exported from STRING. Edge colors denote the type of evidence supporting each interaction. (STRING); Figure S7. PPI network of the targets shared between ginsenoside Rb1 and MASLD. Node size and color intensity are proportional to the degree value of each protein. (Cytoscape); Figure S8. Protein–protein interaction network of the targets shared between ginsenoside Rb1 and MASLD, as exported from STRING. Edge colors denote the type of evidence supporting each interaction. (STRING); Figure S9. PPI network of the targets shared between ginsenoside Rg1 and RA. Node size and color intensity are proportional to the degree value of each protein. (Cytoscape); Figure S10. Protein–protein interaction network of the targets shared between ginsenoside Rg1 and RA, as exported from STRING. Edge colors denote the type of evidence supporting each interaction. (STRING); Figure S11. PPI network of the targets shared between ginsenoside Rb1 and RA. Node size and color intensity are proportional to the degree value of each protein. (Cytoscape); Figure S12. Protein–protein interaction network of the targets shared between ginsenoside Rb1 and RA, as exported from STRING. Edge colors denote the type of evidence supporting each interaction. (STRING); Table S1a. Complete lists of disease targets and their relevance scores for IBD, MASLD and RA; Table S1b. Predicted compound targets for ginsenosides Rg1 and Rb1; Table S2. Evidence levels for the microbiota-mediated effects of ginsenosides Rg1 and Rb1 across IBD, MASLD and RA.

## Abbreviations

AHR, aryl hydrocarbon receptor; AMPK, AMP-activated protein kinase; BA, bile acid; CD, Crohn’s disease; CK, compound K; DMARDs, disease-modifying antirheumatic drugs; DSS, dextran sodium sulfate; FMT, Fecal microbiota transplantation; FXR, Farnesoid X receptor; GPER, G protein-coupled estrogen receptor; HFD, high-fat diet; IAA, indole-3-acetic acid; IAld, indole-3-carboxaldehyde; IBD, inflammatory bowel disease; IFN-γ, interferon-gamma; IL, interleukin; ILA, indole-3-lactic acid; IPA, indole-3-propionic acid; JAK, Janus kinase; LPS, lipopolysaccharide; MAPK, mitogen-activated protein kinase; MASLD, metabolic dysfunction-associated steatotic liver disease; MASH, metabolic dysfunction-associated steatohepatitis; MMP, matrix metalloproteinase; NAFLD, non-alcoholic fatty liver disease; NF-κB, nuclear factor kappa-B; NICD, Notch1 intracellular domain; NLRP3, NOD-like receptor thermal protein domain associated protein 3; PD, pharmacodynamics; PK, pharmacokinetics; PPI, protein–protein interaction; PI3K, phosphoinositide 3-kinase; PPARγ, peroxisome proliferator-activated receptor γ; PPT, protopanaxatriol; PRELP, proline/arginine-rich end leucine-rich repeat protein; RA, rheumatoid arthritis; RANK, receptor activator of nuclear factor-κ B; RANKL, receptor activator of nuclear factor-κ B ligand; SCFA, short-chain fatty acids; SEA, similarity ensemble approach; TCM, Traditional Chinese medicine; Tfh, T follicular helper; Tfr, T follicular regulatory; TLR, toll-like receptor; TNBS, 2,4,6-trinitrobenzene sulfonic acid; TNF-α, tumor necrosis factor-alpha; Treg, regulatory T cell; UC, ulcerative colitis; UCP, uncoupling protein; ZO-1, zonula occludens-1.
