# Supplementary material for: Immunomodulatory and Metabolic Effects of Ginsenosides Rg1/Rb1 in IBD, RA, and MASLD: Targeting Gut Microbiota–Autoimmunity Axis
Source: Microorganisms. 2026 Oct 3;14(10):2246. doi: 10.3390/microorganisms14102246 (PMC13707669; doi:10.3390/microorganisms14102246)
Supplement: Supplementary file 1 [file microorganisms-14-02246-s001.zip › Supplementary Table S2.pdf]

Supplementary Table S2. Evidence levels for the microbiota-mediated effects of ginsenosides Rg1 and Rb1 across IBD, MASLD and RA.

Compound	Disease	Key microbiota-related finding	Evidence level	Supporting approach (reference)
Rg1	IBD	Enrichment of beneficial taxa (*Lactobacillus*, *Muribaculaceae*, *Akkermansia*) and reduction of pro-inflammatory taxa (*Escherichia–Shigella*, *Bacteroides*); increased tryptophan-derived metabolites (IPA, ILA, IAld)	Direct + Indirect	16S rRNA profiling; metabolomics (21, 24, 27)
Rb1	IBD	Increased *Akkermansia* and SCFA-producing taxa; reduced pathogenic taxa; altered pharmacokinetics associated with microbial shifts	Indirect	16S rRNA profiling; PK correlation (91, 97, 100)
Rg1	MASLD	Ameliorates hepatic injury via the gut microbiota–tryptophan–AHR/IL-22 axis; FMT from Rg1-treated donors recapitulates the benefit	Direct	FMT (111)
Rb1	MASLD	Increased *Akkermansia*, *Lactobacillus*, *Allobaculum* and SCFA producers; decreased *Bacteroides*, *Odoribacter*, *Turicibacter*	Indirect	HFD-induced obesity model (112, 113)
Rg1	RA	Restoration of SCFA-producing commensals	Indirect (extrapolated)	No RA-specific interventional study reported to date
Rb1	RA	Possible modulation of immune dysregulation via microbiota	Prediction (extrapolated)	No RA-specific interventional study reported to date
Both	All three diseases	Drug–host target–gut microbiota–microbial	Computational prediction	gutMGene; SEA/TargetNet

	metabolite–host target networks	
