# Supplementary material for: Immunomodulatory and Metabolic Effects of Ginsenosides Rg1/Rb1 in IBD, RA, and MASLD: Targeting Gut Microbiota–Autoimmunity Axis
Source: Microorganisms. 2026 Oct 3;14(10):2246. doi: 10.3390/microorganisms14102246 (PMC13707669; doi:10.3390/microorganisms14102246)
Supplement: Supplementary file 1 [file microorganisms-14-02246-s001.zip › Supplementary_Figure_Legends.pdf]

Supplementary Figure Legends

Sources of Figures

All figures in the Supplementary Material were generated using the software and data resources listed below. Version numbers, URLs, and access dates are stated here once and are not repeated in the individual legends; the bracketed term at the end of each legend indicates the software used to generate that figure.

- **Protein–protein interaction data:** STRING database (version 12.0; https://string-db.org/; accessed on 15 September 2025); Homo sapiens was selected as the organism and a minimum required interaction score of 0.4 was applied.
- **Network construction, topological analysis, and rendering:** Cytoscape (version 3.10.3; Cytoscape Consortium, San Diego, CA, USA). Hub targets were ranked with the cytoHubba plugin (version 0.1) using MNC, Degree, EPC, Betweenness, and Closeness.
- **Venn diagrams and related graphs:** WeiShengXin online platform for data analysis and visualization (https://www.bioinformatics.com.cn/; accessed on 15 September 2025).

Figures S1, S3, S5, S7, S9, and S11 were rendered in Cytoscape from STRING-derived interaction data, with node size and color intensity scaled to the degree value. Figures S2, S4, S6, S8, S10, and S12 were exported directly from the STRING web interface, in which edge colors denote the type of evidence supporting each interaction.

Figure Legends

Supplementary Figure S1. Protein–protein interaction (PPI) network of the targets shared between ginsenoside Rg1 and IBD. Node size and color intensity are proportional to the degree value of each protein. (Cytoscape)

Supplementary Figure S2. Protein–protein interaction network of the targets shared between ginsenoside Rg1 and IBD, as exported from STRING. Edge colors denote the type of evidence supporting each interaction. (STRING)

Supplementary Figure S3. PPI network of the targets shared between ginsenoside Rb1 and IBD. Node size and color intensity are proportional to the degree value of each protein. (Cytoscape)

Supplementary Figure S4. Protein–protein interaction network of the targets shared between ginsenoside Rb1 and IBD, as exported from STRING. Edge colors denote the type of evidence supporting each interaction. (STRING)

Supplementary Figure S5. PPI network of the targets shared between ginsenoside Rg1 and MASLD. Node size and color intensity are proportional to the degree value of each protein. (Cytoscape)

Supplementary Figure S6. Protein–protein interaction network of the targets shared between ginsenoside Rg1 and MASLD, as exported from STRING. Edge colors denote the type of evidence supporting each interaction. (STRING)

Supplementary Figure S7. PPI network of the targets shared between ginsenoside Rb1 and MASLD. Node size and color intensity are proportional to the degree value of each protein. (Cytoscape)

Supplementary Figure S8. Protein–protein interaction network of the targets shared between ginsenoside Rb1 and MASLD, as exported from STRING. Edge colors denote the type of evidence supporting each interaction. (STRING)

Supplementary Figure S9. PPI network of the targets shared between ginsenoside Rg1 and RA. Node size and color intensity are proportional to the degree value of each protein. (Cytoscape)

Supplementary Figure S10. Protein–protein interaction network of the targets shared between ginsenoside Rg1 and RA, as exported from STRING. Edge colors denote the type of evidence supporting each interaction. (STRING)

Supplementary Figure S11. PPI network of the targets shared between ginsenoside Rb1 and RA. Node size and color intensity are proportional to the degree value of each protein. (Cytoscape)

Supplementary Figure S12. Protein–protein interaction network of the targets shared between ginsenoside Rb1 and RA, as exported from STRING. Edge colors denote the type of evidence supporting each interaction. (STRING)
